# Management of Adverse Events in Cancer Patients Treated With PD-1/PD-L1 Blockade: Focus on Asian Populations

**DOI:** 10.3389/fphar.2019.00726

**Published:** 2019-07-02

**Authors:** Jiqiao Yang, Xiujing He, Qing Lv, Jing Jing, Hubing Shi

**Affiliations:** ^1^Laboratory of Tumor Targeted and Immune Therapy, Clinical Research Center for Breast, State Key Laboratory of Biotherapy, West China Hospital, Sichuan University, Chengdu, China; ^2^Clinical Research Center for Breast, State Key Laboratory of Biotherapy, West China Hospital, Sichuan University, Chengdu, China; ^3^Department of Breast Surgery, West China Hospital, Sichuan University, Chengdu, China

**Keywords:** programmed cell death protein 1, programmed death-ligand 1, adverse event, Asian, cancer, immunotherapy

## Abstract

The interaction between programmed cell death protein 1 (PD-1) and its ligand programmed death-ligand 1 (PD-L1) induces exhaustions of cytotoxic lymphocytes in the tumor microenvironment, which facilitates tumor immune evasion. PD-1/PD-L1 blockade therapy, which prevents the receptors and ligands from binding to each other, disrupts the T-cell exhaustion signaling, thereby increasing antitumor immunity. Inspiringly, it has revolutionized the treatment of many different types of cancers including non-small-cell lung carcinoma, melanoma, lymphoma, and so on. However, with the intention of generating an antitumor immune response, PD-1/PD-L1 blockade may also lead to a spectrum of side effects. The profile of adverse events (AEs) of PD-1/PD-L1 blockade is not exactly the same with other immune checkpoint blockades, such as blockade of cytotoxic T-lymphocyte-associated protein 4. Although cutaneous, gastrointestinal, and pulmonary systems are common victims, AEs of PD-1/PD-L1 blockade might occur in any other organ system of the human body. These toxicities can be life-threatening if not managed promptly, and proper treatment intervention is imperative for optimal control and prevention of severe damage. Currently, clinical practice for the management of AEs in PD-1/PD-L1 blockade remains sporadic and variable. The majority of initial clinical trials were carried out in Caucasians. The trials of multiple races usually included a small portion of Asian participants, and results were calculated and interpreted for the entire included subjects without any race-specific conclusions. Therefore, the information on PD-1/PD-L1 blockade in Asians is far from systematic or comprehensive. Recently, as the results of clinical trials of anti-PD-1/PD-L1 agents in Asian populations have been gradually released, we summarized current evidence with a specific focus on the Asian population, hoping to outline strategies and offer guidance on the management of AEs in cancer patients treated with PD-1/PD-L1 blockade in the Asian world.

## Background

### Overview of Programmed Cell Death Protein 1/Programmed Death-Ligand 1 Blockade

**Figure 1:**
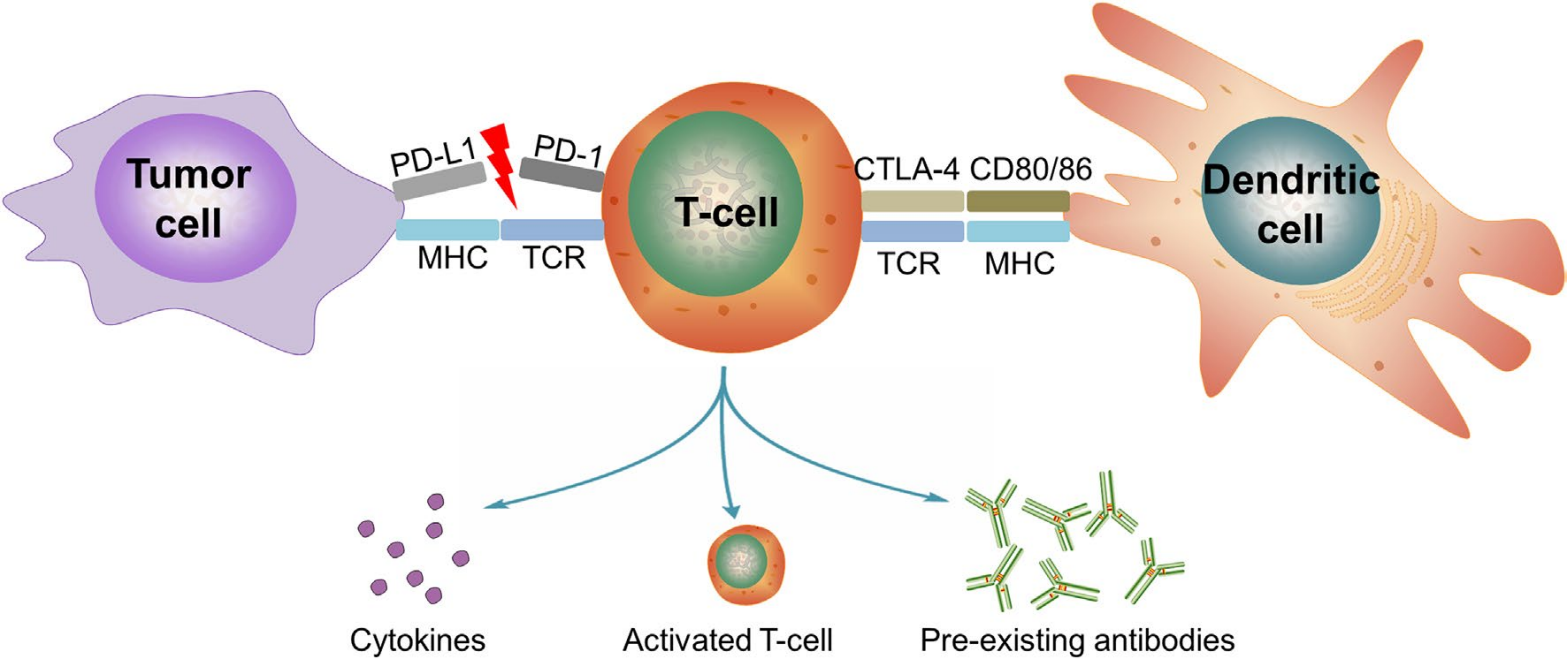
Possible mechanisms of immune-related adverse events in cancer patients treated with PD-1/PD-L1 blockade. PD-L1 is expressed in tumor cells. After prolonged activation, PD-1 is upregulated in T cells and binds to its ligands on tumor cells or other immune cells to dampen an ongoing immune response. Anti-PD-1/PD-L1 therapy blocks this inhibitory signaling, thereby provoking the immune response to tumor. Possible mechanisms of immune-related adverse events with PD-1/PD-L1 blockade include 1) off-target effects of T cell-mediated immunity in healthy tissue, such as in myocarditis and pneumonitis; 2) increased preexisting autoantibodies, such as in arthritis and thyroid toxicity; and 3) increased inflammatory cytokines (Calabrese et al., 2018; Postow et al., 2018). (PD-1, programmed cell death protein 1; PD-L1, programmed death-ligand 1; CTLA-4, cytotoxic T-lymphocyte-associated protein 4; TCR, T-cell receptor; MHC, major histocompatibility complex).

Programmed cell death protein 1 (PD-1), also known as cluster of differentiation 279 (CD279), is a protein expressed on the surface of cells. The principal ligand of PD-1, programmed death-ligand 1 (PD-L1), also known as B7-H1 or CD274 (Ishida et al., 1992), is frequently expressed within the tumor microenvironment, including in cancer cells, antigen presenting cells (APCs), tumor-infiltrating macrophages, T cells, B cells, dendritic cells, and mesenchymal stem cells (Weber, 2010; Pardoll, 2012). Interacting with its cell surface ligands, PD-1 negatively regulates the effector phase of T-cell responses (Blank et al., 2004) (**Figure 1**). Through multiple mechanisms including simultaneous proapoptotic effects in cytotoxic T cells and antiapoptotic effects in regulatory T cells, PD-1 downregulates the immune system and promotes self-tolerance. This regulates the immune system’s response to the cells and prevents the immune system from killing tumor cells in the human body (Syn et al., 2017).

Immune checkpoint inhibitors (ICIs) blocking the interaction of PD-1 and PD-L1 significantly enhance T-cell function and therefore exert antitumor activity (Brahmer et al., 2012). By now, several anti-PD-1/PD-L1 antibodies have been developed, including nivolumab, pembrolizumab, cemiplimab, and camrelizumab (anti-PD-1 antibodies) as well as atezolizumab, durvalumab, and avelumab (anti-PD-L1 antibodies). The efficacies of these anti-PD-1/PD-L1 agents have been proven across various cancer types, such as melanoma (Hamid et al., 2013; Robert et al., 2015; Weber et al., 2015b), non-small-cell lung cancer (NSCLC) (Nishio et al., 2017), and Hodgkin lymphoma (Maruyama et al., 2017). Several agents have been approved by the US Food and Drug Administration (FDA) and the European Medicines Agency because of their great performance over conventional treatments in malignancies.

### Adverse Events in Cancer Patients Treated With Programmed Cell Death Protein 1/Programmed Death-Ligand 1 Blockade

Immune checkpoints are an essential component of the immune system. They function in a delicate organism of self-regulation to avoid excessively activated or even deleterious immune responses (Postow et al., 2012). Among the immune checkpoints, the PD-1/PD-L1 pathway is a crucial regulator in balancing the activation and tolerance of T cells (Okazaki and Honjo, 2006). The basic idea of PD-1/PD-L1 blockade is to block the interaction of PD-1 on T cells and PD-L1 on tumor cells, which offers tumor cells additional resistance to T-cell-mediated apoptosis, thus preventing cancer cells from defending themselves against antitumor immune responses (Azuma et al., 2008).

However, PD-L1 also exists in noncancer tissues, such as pancreatic islets, heart, endothelium, small intestine, and many other tissues yet to be discovered (Simeone and Ascierto, 2012). In preclinical researches, PD-1-deficient mice exhibited systemic lupus erythematosus-like disease (Nishimura et al., 1999), lupus-like arthritis, glomerulonephritis (Nishimura et al., 1999), and cardiomyopathy (Nishimura et al., 2001). Moreover, the polymorphism in PD-1 has been associated with autoimmune diseases in humans (Prokunina et al., 2002). The above evidence indicated that blockade of the PD-1/PD-L1 pathway may induce autoimmune disease and systemic inflammation (**Figure 1**). As confirmed in clinical trials, a spectrum of immune-related adverse events (irAEs) has emerged because of the turbulence in immunomodulation accompanying PD-1/PD-L1 blockade (Postow et al., 2018), despite favorable efficacy in suppressing tumors.

### Uniqueness of Immune-Related Adverse Events in Cancer Patients Treated With Programmed Cell Death Protein 1/Programmed Death-Ligand 1 Blockade

IrAEs are defined as any AE associated with exposure to immunotherapy and with an immune-mediated mechanism. Upon the diagnosis of an irAE, infections and other definite etiologies should be ruled out (Sgambato et al., 2016). Anti-cytotoxic T-lymphocyte-associated protein-4 (CTLA-4) and anti-PD-1/PD-L1 agents are both ICIs that form the new generation of immunotherapy and share a similar background in drug development. Nevertheless, the profile of AEs in patients treated with PD-1/PD-L1 blockade is not exactly the same with those treated with other ICIs such as CTLA-4 blockade.

PD-1 bears homology to CTLA-4 but provides distinct immune-inhibitory signals. More specifically, PD-1 impedes the activity of effector T cells in the effector phase, whereas CTLA-4 regulates T-cell function in an earlier activation phase. Moreover, PD-1 is expressed in various types of cells including T cells, B cells, natural killer cells, and macrophages. Unlike PD-1, the expression of CTLA-4 is confined to T cells (Dong et al., 2002; Fanoni et al., 2011; Ribas, 2012). As described previously, the PD-1 receptor is crucially involved in peripheral tolerance, PD whereas CTLA-4 is pivotal in central tolerance and control (Sharon et al., 2014).

In preclinical animal models, the autoimmune phenotypes were different between PD-1-deficient mice and CTLA-4-deficient mice (Nishimura et al., 1999, Nishimura et al., 2001). In the PD-1 knockout mice, strain- and organ-specific autoimmunity was demonstrated in a modest later-onset model compared with early lethality in CTLA-4 knockout mice (Nishimura et al., 1999, Nishimura et al., 2001). As shown in clinical trials, PD-1/PD-L1 blockade is associated with a different spectrum of irAEs from anti-CTLA-4 therapy. As an example, nivolumab has been associated with a unique spectrum of pneumonitis (Postow et al., 2012). Another example is that colitis is more frequently seen in patients treated with ipilimumab (CTLA-4 blockade) than in patients treated with anti-PD-1 therapy (Agarwala, 2015).

Although PD-1/PD-L1 blockade reveals comparatively fewer and milder toxic effects than those for CTLA-4 blockade (Brahmer et al., 2010; Ribas, 2012; Zumelzu et al., 2018), the definite incidence of AEs in patients with PD-1/PD-L1 blockade was high, and some high-grade AEs can be lethal. Therefore, the current review aimed to summarize the recent updates in the management of AEs in patients under PD-1/PD-L1 blockade. Up to now, majority of AE experience in patients treated with ICIs comes from clinical trials in the Western world. As phased results of clinical trials of PD-1/PD-L1 blockade in Asians are being published, the current review focuses on the profile of the Asian world in the field of the management of AEs.

## Adverse Events of Programmed Cell Death Protein 1/Programmed Death-Ligand 1 Blockade in Asian Populations

**Figure 2:**
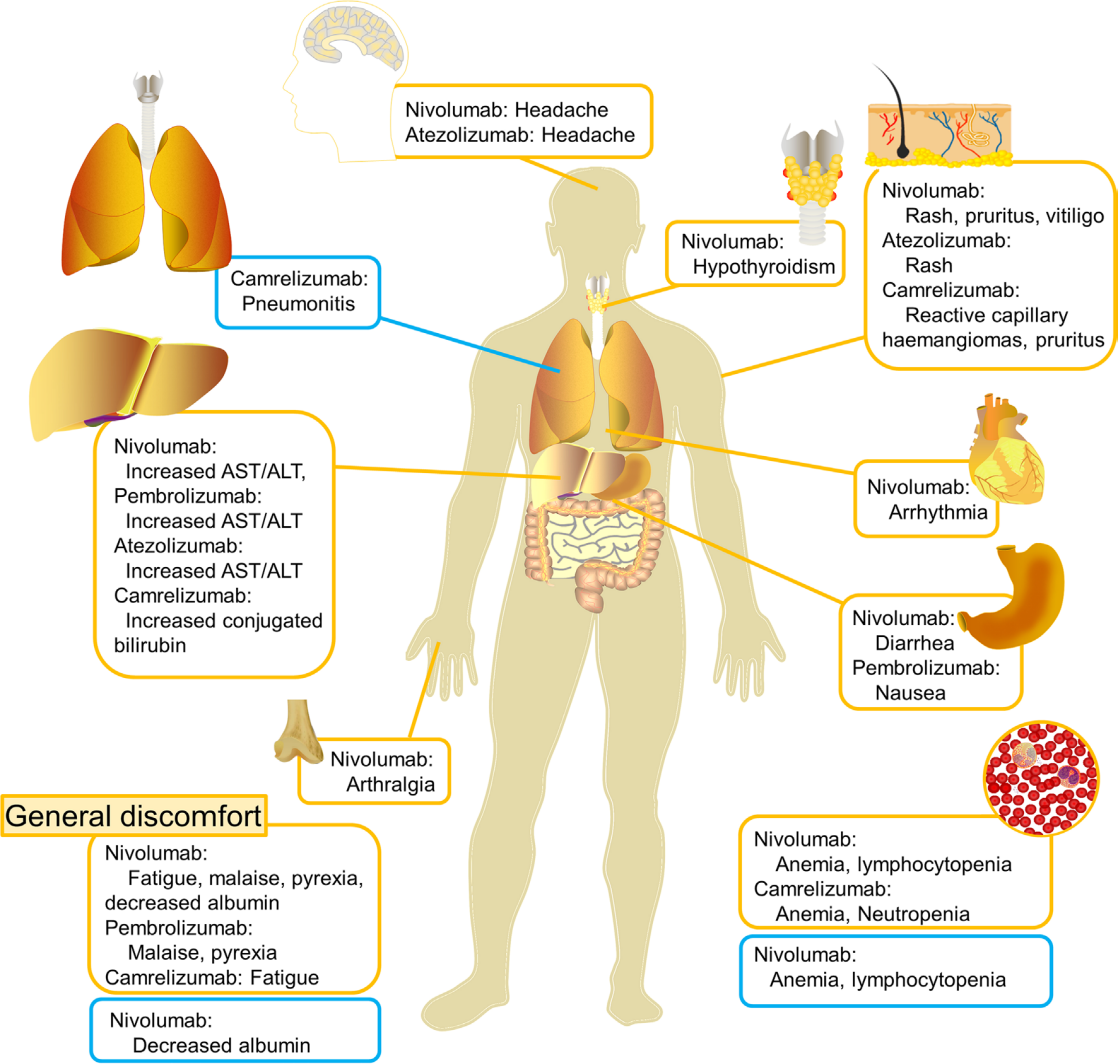
Clinical spectrum of treatment-related adverse events reported in clinical trials of major PD-1/PD-L1 blockade in Asian populations. The most common adverse events (AEs) of any grade per organ system were dermatological toxicities, hepatic toxicities, endocrinopathies, and general disorders. The incidence of pulmonary toxicities was lower, but it is the most common reason for a serious AE. (Orange text boxes: treatment-related AEs of any grade observed in ≥20% of patients in clinical trials; blue text boxes: treatment-related AEs of grades 3–5 observed in ≥5% of patients in clinical trials, unless a case of onset <2 in trials with a small sample size; AST, aspartate aminotransferase; ALT, alanine aminotransferase.)

**Figure 3:**
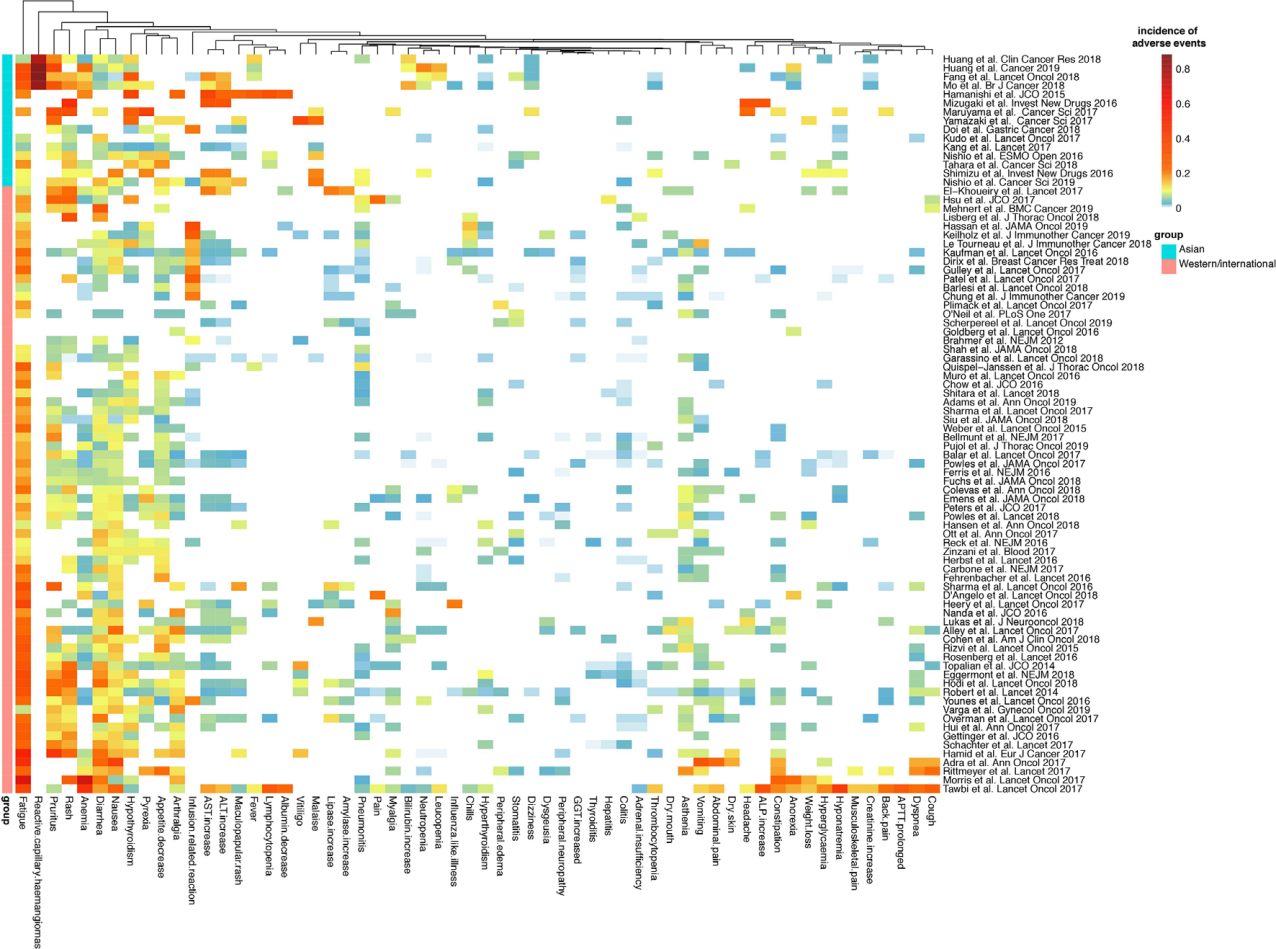
Heatmap of the incidence of the top 60 most common AEs of any grade in cancer patients treated with PD-1/PD-L1 blockade. Fifteen clinical trials in Asian patients and 69 trials in Western or mixed international population of PD-1/PD-L1 blockade monotherapy (including nivolumab, pembrolizumab, atezolizumab, avelumab, durvalumab, and camrelizumab) were included. APTT, activated partial thromboplastin time; ALP, alkaline phosphatase; GGT, gamma-glutamyltransferase; ALT, alanine aminotransferase; AST, aspartate aminotransferase.)

**Figure 4:**
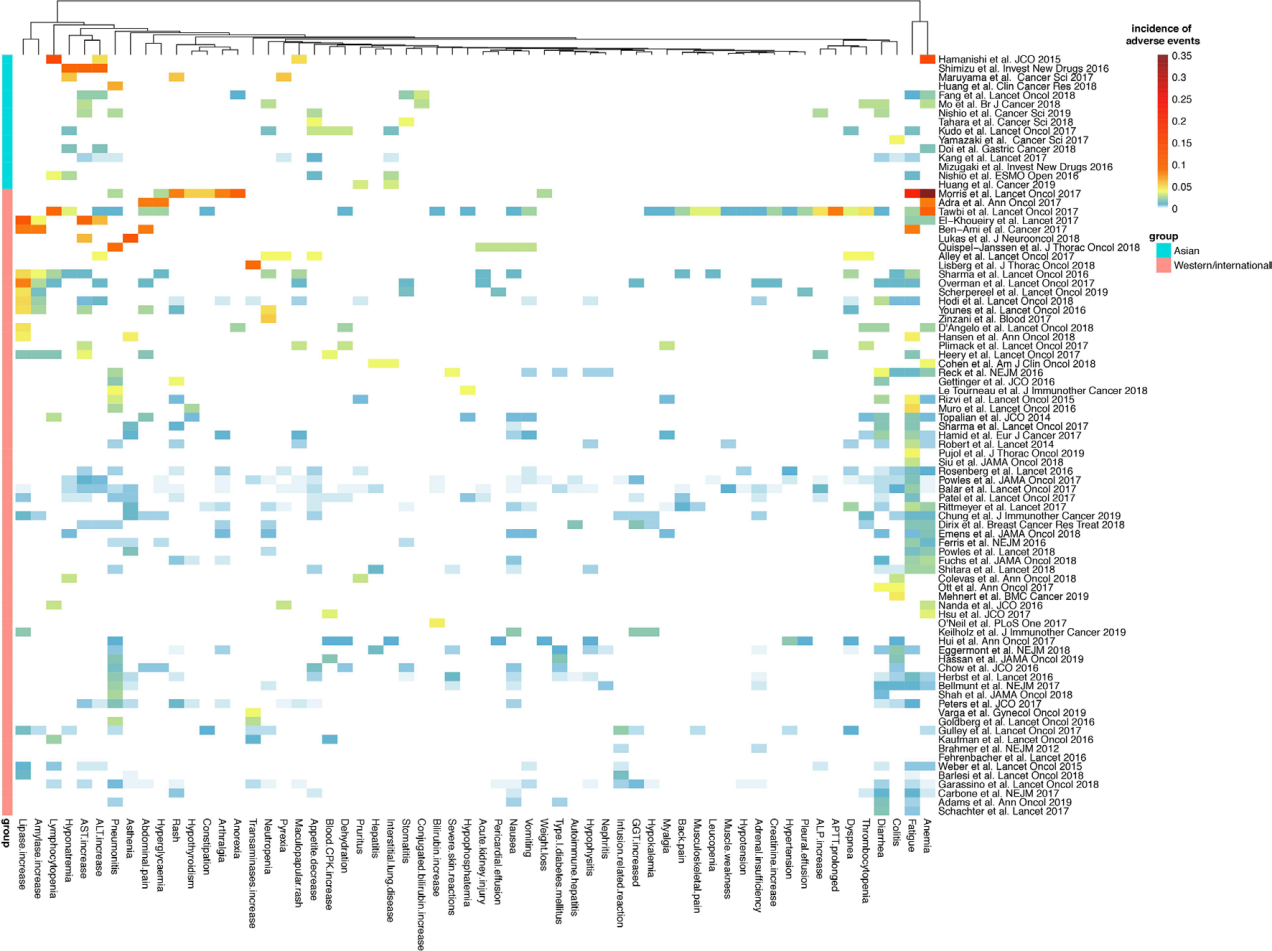
Heatmap of the incidence of the top 60 most common AEs of grades 3–5 in cancer patients treated with PD-1/PD-L1 blockade. Fifteen clinical trials in Asian patients and 70 trials in Western or mixed international population of PD-1/PD-L1 blockade monotherapy (including nivolumab, pembrolizumab, atezolizumab, avelumab, durvalumab, and camrelizumab) were included. (APTT, activated partial thromboplastin time; ALP, alkaline phosphatase; GGT, gamma-glutamyltransferase; ALT, alanine aminotransferase; AST, aspartate aminotransferase; CPK, creatinine phosphokinase.)

**Figure 5:**
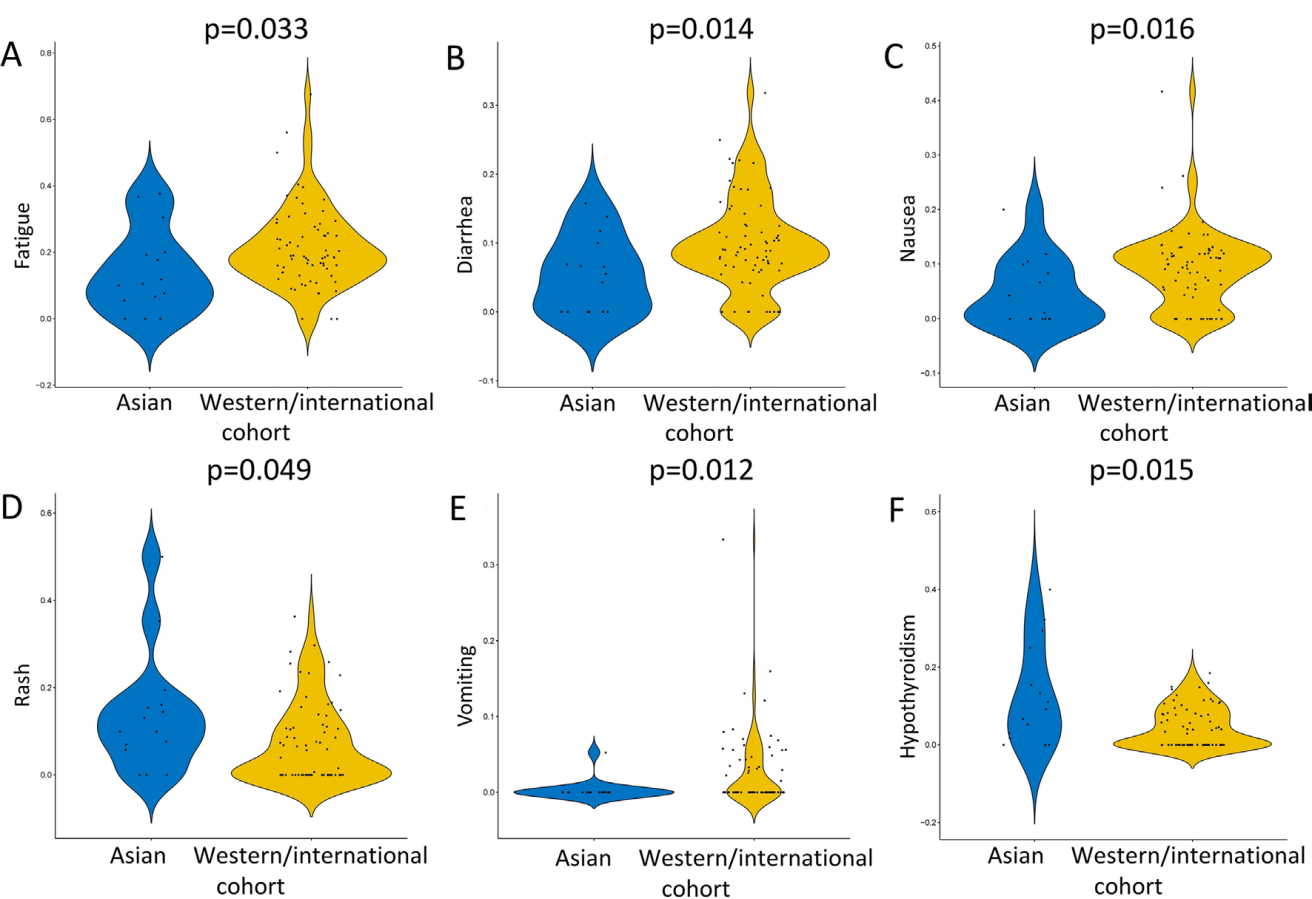
Selected adverse events with different incidences between Asian populations and Western/international populations in cancer patients treated with PD-1/PD-L1 blockade. The adverse events (AEs) of any grade with different prevalences between Asian populations and Western/international populations include fatigue **(A)**, diarrhea **(B)**, nausea **(C)**, rash **(D)**, vomiting **(E)**, hypothyroidism **(F)**, ALT increase, asthenia, dizziness, fever, adrenal insufficiency, hyponatremia, lipase, malaise, and reactive capillary hemangiomas. The AEs of grades 3–5 with different prevalences between Asian populations and Western/international populations include fatigue, nausea, interstitial lung disease, lipase increase, hyponatremia, and increase in conjugated bilirubin. The comparative analysis was only performed in AEs with at least one event in both Asian patients and Western/international patients.

Among the PD-1/PD-L1 inhibitors, nivolumab, pembrolizumab, atezolizumab, avelumab, and durvalumab have been approved by the FDA in the United States for the treatment of cancers. The majority of initial clinical trials were carried out in Caucasians. Large multicenter trials with patients of mixed races usually included a small portion of Asian participants, and results such as response rate, survival, and incidence of AEs were calculated and interpreted for the entire included subjects. Therefore, the information of PD-1/PD-L1 blockade in Asians is far from systematic or comprehensive. By now, the results of trials on nivolumab (Hamanishi et al., 2015; Kang et al., 2017; Kudo et al., 2017; Maruyama et al., 2017; Nishio et al., 2017; Yamazaki et al., 2017), camrelizumab (Fang et al., 2018; Huang et al., 2018, Huang et al., 2019; Mo et al., 2018), pembrolizumab (Shimizu et al., 2016; Tahara et al., 2018; Nishio et al., 2019), avelumab (Doi et al., 2018), and atezolizumab (Mizugaki et al., 2016) in Asian populations have been published (**Table 1**). Here, the most common treatment-related AEs (TRAEs) of any grade and TRAEs of grades 3–5 reported in the above articles were summarized per organ system (**Figure 2**). To explore whether the profile of AEs in Asian patients is similar to those in studies carried out in Caucasians or mixed races, we searched the Pubmed database and extracted the incidence of each reported AE from available results of clinical trials (**Supplementary Table 1**). The search terms “(PD-L1 OR) AND trial” were used, and the last search date was April 6, 2019. We also manually screened the references of related studies to avoid omissions. The inclusion criteria of studies include the following: a) clinical trials of cancer patients treated with PD-1/PD-L1 blockade published in English; b) studies reporting the incidence of AEs of any system. Accordingly, clinical trials with null information on the prevalence of AEs and case reports of rare AEs were excluded. Single-center studies with patients of Western origin, multicenter studies of patients from mixed Western origins, and large multicenter trials with patients of mixed races, which included a portion of Asian participants, were classified as “Western/international” studies. Single-center studies with patients of Asian origin and multicenter studies of patients from mixed Asian origins were classified as “Asian” studies. We displayed the top 60 AEs of any grade (**Figure 3**) and AEs of grades 3–5 (**Figure 4**) with a heatmap. The incidence of each AE was compared between Asian and Western/international populations, and selected AEs with significantly different incidences between groups were shown (**Figure 5**). Hierarchical clustering analysis was performed based on the incidence of AEs by using the pheatmap package (https://cran.r-project.org/web/packages/pheatmap/index.html, version 1.0.12). The AEs with different prevalences between Asian and Western/international populations were depicted by violin plots. Statistical analysis was performed using the Wilcoxon test in ggpubr package (https://cran.r-project.org/web/packages/ggpubr/index.html, version 0.2). P values <0.05 were considered statistically significant. The features of AEs in Asian patients treated with PD-1/PD-L1 blockade are discussed per agent below.

**Table 1:** Incidence of AEs in published results of clinical trials of anti-PD-1/PD-L1 monotherapy in Asian populations.

Year	Trial number	Country/Region	Agent	Cancer	Phase	Sample size	Rate of AE	Rate of TRAE	Rate of irAE	Treatment interrupted because of AE	Treatment discontinued because of AE	Common types of AE
2017	JapicCTI-142422 (Kudo et al., 2017)	Japan	Nivolumab	Esophageal carcinoma	2	65	85%	60%	NA	23%	11%	Diarrhea, appetite decrease, constipation
2017	JapicCTI-142533 (Yamazaki et al., 2017)	Japan	Nivolumab	Melanoma	2	24	91.7%	83.3%	NA	8.3%	8.3%	Vitiligo, pruritus, hypothyroidism, malaise
2017	JapicCTI-142755 (Maruyama et al., 2017)	Japan	Nivolumab	Hodgkin lymphoma	2	17	100%	NA	NA	41.2%	NA	Pyrexia, pruritus, rash
2016	JapicCTI-132073 (Nishio et al., 2017)	Japan	Nivolumab	NSCLC	2	76	NA	84.2%	NA	NA	15.8%	Malaise, pyrexia, rash, appetite decrease
2015	UMIN000005714 (Hamanishi et al., 2015)	Japan	Nivolumab	Ovarian cancer	2	20	NA	95%	NA	NA	11%	AST increase, hypothyroidism, lymphocytopenia
2017	NCT02267343 (Kang et al., 2017)	Japan, South Korea, Taiwan	Nivolumab	Gastric and gastroesophageal junction cancer	3	330	91%	43%	NA	NA	2.7%	Pruritus, diarrhea, rash, fatigue
2018	NCT02721589 (Fang et al., 2018)	China	Camrelizumab	Nasopharyngeal carcinoma	1	93	NA	97%	NA	12.9%	2.2%	Reactive capillary hemangiomas, fatigue, hypothyroidism
2018	NCT02742935 (Mo et al., 2018)	China	Camrelizumab	Solid tumors	1	36	97.2%	88.9%	86.1%	NA	2.8%	Reactive capillary hemangiomas, pruritus, fatigue
2018	NCT02742935 (Huang et al., 2018)	China	Camrelizumab	Esophageal carcinoma	1	30	NA	83.3%	83.3%	6.7%	0	Reactive capillary hemangiomas, pruritus, hypothyroidism
2019	NCT02742935 (Huang et al., 2019)	China	Camrelizumab	Gastric and gastroesophageal junction cancer	1	30	100%	100%	93.3%	NA	NA	Reactive capillary hemangiomas, pruritus, fatigue
2016	NCT01840579 (Shimizu et al., 2016)	Japan	Pembrolizumab	Solid tumors	1	10	NA	80%	40%	NA	0	Nausea, malaise, pyrexia
2018	NCT02007070 (Nishio et al., 2019)	Japan	Pembrolizumab	NSCLC	1b	38	NA	87%	24%*	NA	11.1%	Malaise, diarrhea, maculopapular rash
2018	NCT01848834 (Tahara et al., 2018)	Japan, South Korea, Taiwan	Pembrolizumab	Head and neck squamous cell carcinoma	1b	26	NA	62%	19%	NA	3.8%	Fatigue, appetite decrease, hypothyroidism, rash
2016	JapicCTI-132208 (Mizugaki et al., 2016)	Japan	Atezolizumab	Solid tumors	1	6	100%	NA	NA	50%	0	Rash, increased AST, ALT, and ALP, headache
2018	NCT01943461 (Doi et al., 2018)	Japan	Avelumab	Solid tumors	1	17 (dose-escalation cohort)	94.1%	64.7%	11.8%	NA	0	Infusion-related reaction, rash maculopapular, stomatitis
2018	NCT01943461 (Doi et al., 2018)	Japan	Avelumab	Solid tumors	1	40 (dose-expansion cohort)	100%	80%	12.5%	NA	10%	Infusion-related reaction, pruritus, pyrexia
2019	NCT02836795 (Tang et al., 2019)	China	Toripalimab	Melanoma and urologic cancer	1	36	100%	100%	NA	16.7%	14%	Hyperglycemia, proteinuria, rash
2019	NCT03114683 (Shi et al., 2019)	China	Sintilimab	Classical Hodgkin lymphoma	2	92	100%	93%	54%	NA	3%	Pyrexia, hypothyroidism, increased TSH

PD-1, programmed cell death protein 1; PD-L1, programmed death-ligand 1; NSCLC, non-small-cell lung cancer; AE, adverse event; TRAE, treatment-related adverse event; irAE, immune-related adverse event; AST, aspartate aminotransferase; ALT, alanine aminotransferase; ALP, alkaline phosphatase; TSH, thyroid-stimulating hormone; NA, not available.

*rate of irAE plus infusion reaction.

### Nivolumab

Nivolumab (BMS-936558/ONO-4538) is a fully human monoclonal immunoglobulin G4 (IgG4) antibody inhibitor of PD-1 (Hardy et al., 1997). Nivolumab has been approved by FDA as monotherapy in unresectable or metastatic melanoma, metastatic NSCLC, advanced renal cell carcinoma, locally advanced or metastatic urothelial carcinoma, recurrent or metastatic head and neck squamous cell carcinoma, relapsed or refractory classical Hodgkin lymphoma, microsatellite instability-high or mismatch repair deficient metastatic colorectal cancer, or hepatocellular carcinoma that has been previously treated with sorafenib and in combination with ipilimumab in unresectable or metastatic melanoma (Bristol-Myers, 2019). In a recent meta-analysis, hypothyroidism, pneumonitis, colitis, and hypophysitis were several of the most common irAEs of any grade. Among them, pneumonitis was the most common serious AE (Baxi et al., 2018).

In Asians, results of phase 2 and 3 trials of nivolumab have been published (Hamanishi et al., 2015; Kang et al., 2017; Kudo et al., 2017; Maruyama et al., 2017; Nishio et al., 2017; Yamazaki et al., 2017). In a phase 2 trial of 65 patients with esophageal squamous cell carcinoma in Japan, the most common AEs were diarrhea, appetite decrease, constipation, rash, and fatigue, the majority of which resolved with drug discontinuation and/or supportive care. Twenty-six percent of patients developed grade 3–4 AEs, and 17% developed serious AEs. Serious AEs that occurred in this group of patients included lung infection, dehydration, and interstitial lung disease (ILD) (Kudo et al., 2017). In a phase 2 trial of nivolumab in Japanese patients with previously untreated advanced melanoma, AEs were reported in 91.7% of patients. Serious AEs that occurred were colitis, abnormal hepatic function, renal impairment, and pleural effusion. The case of colitis was the only TRAE of grade ≥3. These patients experienced two episodes of colitis. The first episode of colitis alleviated with the treatment with corticosteroids and suspension of nivolumab but relapsed 5 months later when nivolumab was discontinued (Yamazaki et al., 2017). As with two other phase 2 trials of nivolumab in Japanese patients with classical Hodgkin lymphoma (Maruyama et al., 2017) and NSCLC (Nishio et al., 2017), no treatment-related death was observed. The results of a phase 3 study of patients with advanced gastric or gastroesophageal junction cancer refractory to, or intolerant of, at least two previous chemotherapy regimens have been recently published (Kang et al., 2017). The study included 330 patients in the nivolumab group and 161 in the placebo group from Japan, South Korea, and Taiwan. TRAEs were observed in 43% of patients in the nivolumab group and 27% of patients in the placebo group. Five (2%) patients in the nivolumab group and two (1%) patients in the placebo group died from TRAE (Kang et al., 2017). Generally, the safety profile of nivolumab in Asians was similar to what was reported in previous large international studies (Robert et al., 2015; Weber et al., 2015b).

### Camrelizumab

Camrelizumab (SHR-1210) is a selective, humanized, high-affinity IgG4-κ monoclonal antibody against PD-1 developed by Jiangsu Hengrui Medicine (Huang et al., 2019). All the trials of camrelizumab with published data were performed in China (Fang et al., 2018; Huang et al., 2018, Mo et al., 2018;Huang et al., 2019).

The results of a large phase 1 clinical trial (NCT02742935) have been reported in esophageal carcinoma (Huang et al., 2018), nasopharyngeal carcinoma (Fang et al., 2018), and gastric cancer (Huang et al., 2019). The antitumor efficacy was promising. Unlike a varied spectrum of AEs in different types of cancers with other PD-1 inhibitors, the most common TRAEs in different cancer types were concentrated in reactive capillary hemangiomas (RCHs), pruritus, hypothyroidism, fatigue, and hypothyroidism. The safety profile of camrelizumab in Asian patients was similar to that of other PD-1 inhibitors (Huang et al., 2018). Although more than 80% of participants experience AEs of any grade, most AEs were grade 1 or 2 and could be well managed with supportive care or medical therapy.

Notably, RCH was seen in a large portion of patients, involving 76.7% of patients with advanced esophageal carcinoma (Huang et al., 2018), 88% of patients with nasopharyngeal carcinoma (Fang et al., 2018), 86.6% of patients with advanced gastric and gastroesophageal junction cancer (Huang et al., 2019), and 83.3% of patients with solid tumors (including NSCLC, breast cancer, colorectal cancer, and hepatocellular carcinoma) (Mo et al., 2018). This phenomenon has never been observed in other PD-1/PD-L1 inhibitors.

RCH is a type of reactive hyperproliferative vascular response. The clinical manifestations of RCH triggered by camrelizumab include red papules or macules with clear boundaries. It can be disseminated all over the body, and the most frequently involved areas are trunks, upper extremities, head, and neck (Huang et al., 2018). RCH emerged at a median time of 23 days after usage of camrelizumab (Mo et al., 2018). A common complication of RCH is bleeding, and a rare complication is ulceration (Teng et al., 2019), which calls for extra supportive care.

Despite the high incidence, the severity of camrelizumab-associated RCH was mostly grade 1, and none of the patients terminated therapy because of this AE. Symptoms of RCH could be spontaneously mitigated during the treatment, but complete regression only occurred after discontinuation of camrelizumab (Huang et al., 2018, Huang et al., 2019).

The mechanism of RCH in patients receiving camrelizumab is yet elucidated. Potential explanations include the imbalance of receptor/receptor–ligand interactions with upregulation of vascular proliferative proteins (Piguet and Borradori, 2002; Teng et al., 2019). Another experimental study using human receptor proteome screening indicated that camrelizumab mediated off-target binding to human receptors, such as vascular endothelial growth factor receptor 2 (VEGFR2), which might thereby drive hemangioma development *via* vascular endothelial cell activation (Finlay et al., 2019). Interestingly, RCH was less frequently seen in patients of advanced hepatocellular carcinoma, gastric, or esophagogastric junction cancer who received camrelizumab in combination with apatinib (Xu et al., 2019). Moreover, the incidence of RCH dropped to 22%, and severity was lower in patients treated with a combinatory regimen of camrelizumab and gemcitabine plus cisplatin. This might be explained that the chemotherapy inhibited the hyperproliferation of endothelial cells (Fang et al., 2018). Currently, the phase 3 trial of camrelizumab is ongoing, and the mechanism of this particular AE shall be further explored for potential drug refinement to prevent unwanted properties.

### Pembrolizumab

Pembrolizumab (MK-3475), previously known as lambrolizumab, is a highly selective IgG4-κ humanized isotype monoclonal antibody against PD-1. It is designed to prevent Fc-mediated antibody-dependent cellular cytotoxicity, thus avoiding cytotoxic effects of the antibody when it binds to the T cells (Hamid et al., 2013).

In a phase 1 trial of pembrolizumab in the treatment of 10 Japanese patients with advanced solid tumors including NSCLC, melanoma, and breast cancer, grade 3 alanine transaminase (ALT) elevation, grade 3 aspartate transaminase (AST) elevation, grade 1 pneumonitis, and grade 1 thyroid-stimulating hormone (TSH) elevation were reported as irAEs (Shimizu et al., 2016). One patient with advanced NSCLC developed grade 3 ALT elevation, grade 3 AST elevation, and grade 1 pneumonitis simultaneously on day 42 and further developed grade 3 hyponatremia after termination of pembrolizumab (Shimizu et al., 2016). In a phase 1b study (KEYNOTE-012) in Asia-Pacific patients with advanced head and neck squamous cell carcinoma, two (8%) patients experienced serious TRAEs, one of which was a grade 2 ILD that resulted in drug discontinuation (Tahara et al., 2018).

As seen from published data, the safety profile of pembrolizumab in Asian populations is generally similar to that in non-Asian patients (Shimizu et al., 2016). However, results of large trials are needed to validate the conclusion, considering the small sample size of existing trials in Asians.

### Other Programmed Cell Death Protein 1/Programmed Death-Ligand 1 Blockades

Among various anti-PD-1/PD-L1 agents, another two agents with published data of trials in Asians are atezolizumab and avelumab (Mizugaki et al., 2016; Doi et al., 2018). Atezolizumab (MPDL3280A) is a human IgG1 monoclonal anti-PD-L1 antibody. Because it does not block the interaction of PD-1 and its second ligand PD-L2, the immune homeostasis is maintained theoretically (Chen et al., 2012). In a phase 1 study of monotherapy with atezolizumab in Japanese patients with advanced solid tumors, all six patients experienced AEs, and half of the patients developed AEs that led to suspension of atezolizumab, including influenza-like illness and increased alkaline phosphatase. Still, all events were grade 1 or 2, and no death occurred (Mizugaki et al., 2016).

Avelumab, another human IgG1 monoclonal anti-PD-L1 antibody, has also been tested in Asian populations. In a phase 1 trial of avelumab in Japanese patients with advanced solid tumors, the most common AEs were infusion-related reactions (IRRs) and rash in the dose-escalation cohort and IRRs and pruritus in the dose-expansion cohort (Doi et al., 2018). In more recent phase 1b studies in Europe and the United States, less cutaneous but more general toxicities such as fatigue, chills, and diarrhea were observed. Nevertheless, IRRs remain the dominant AE across populations (Disis et al., 2019; Hassan et al., 2019).

Apart from the above, the safety profiles of novel agents such as sintilimab (Shi et al., 2019) and toripalimab (JS001) (Tang et al., 2019) have also been reported in Asians. Moreover, a number of large trials of anti-PD-1/PD-L1 monotherapy or combinatory therapy with chemotherapy and/or targeted therapy are ongoing. A growing body of evidence is expected to contribute to the profile of AEs of PD-1/PD-L1 blockade in Asian populations.

## Management of Immune-Related Adverse Events in Cancer Patients Treated With Programmed Cell Death Protein 1/Programmed Death-Ligand 1 Blockade

The incidence of any grade irAEs in clinical trials in Asian populations reportedly is as low as 12% to as high as greater than 90% (Doi et al., 2018; Huang et al., 2019). For some PD-1 inhibitors, the frequency, but not the type, of irAE may increase with dose (Barbee et al., 2015). The profile of irAEs varies among different types of malignancies. A possible explanation is that the irAEs may be associated with the sites of action or sites with T-cell aggregation (Barbee et al., 2015).

In clinical trials, the severity of AEs was evaluated and reported using the Common Terminology Criteria for Adverse Events, which grades AEs on a scale of 1 for mild events that do not need intervention to 5 for death related to the AE (U.S. Department of Health and Human Services, 2018). Although precise practice protocols vary with irAE and anti-PD-1/PD-L1 agent, the American Society of Clinical Oncology has provided general recommendations for irAEs with ICI therapy: for grade 1 AEs, continue therapy with close monitoring; for grade 2 AEs, suspend the therapy and consider resuming when symptoms and/or laboratory values revert to grade ≤1. Corticosteroids may be administered as appropriate; for grade 3 AEs, suspend the therapy and initiate high-dose corticosteroids. If symptoms do not improve within 2–3 days, infliximab may be offered as appropriate; for grade 4 AEs, permanently discontinue the therapy, with the exception of endocrinopathies that have been controlled by hormone replacement (Brahmer et al., 2018). AEs related to PD-1/PD-L1 blockade are generally of low grade (grade 1–2) (Lu et al., 2015). With prompt and proper management, most grade 1–2 AEs can be resolved within a relatively short time (Brennan et al., 2010). However, serious AEs can always be fatal. Therefore, close and continuous monitoring, early recognition, and proper intervention of AEs with rapid onset and poor outcomes are paramount for clinical management. Patient/family member education on self-monitoring should also be involved (Champiat et al., 2015). Currently, prophylaxis against irAEs is not routinely recommended (Barbee et al., 2015).

Because irAEs with PD-1/PD-L1 blockade affect a wild spectrum of body systems, the management of these toxicities requires the collaborative efforts of a multidisciplinary team, including oncologists, pathologists, radiologists, dermatologists, endocrinologists, pulmonologists, neurologists, rheumatologists, gastroenterologists, and the nursing team (Brahmer et al., 2018).

### Pulmonary Toxicity

Pneumonitis is the leading pulmonary toxicity among irAEs with ICI treatment. PD-1/PD-L1 blockade-related pneumonitis is caused by off-target effects against the normal lung parenchyma. In a real-world retrospective study of nivolumab/pembrolizumab monotherapy in Asian patients with NSCLC, grade 4 pneumonitis with subsequent mortality was the most serious AE, which occurred in 3.8% (3/74) of patients (Lin et al., 2018). In another retrospectively study of 123 patients with NSCLC treated with nivolumab or pembrolizumab in Japan, 18 patients (14.6%) experienced anti-PD-1-related pneumonitis, of which four (3.3%) were grade ≥3 (Jodai et al., 2018). It has been observed in less than 10% of patients receiving PD-1/PD-L1 inhibitors, but it can quickly escalate and is one of the major causes of treatment-related death (Naidoo et al., 2015). Compared with PD-1 inhibitors, severe pneumonitis is less seen with PD-L1 inhibitors (Kong and Flynn, 2014). Of the two ligands of PD-1, PD-L1 is distributed in a broad spectrum of tissues, whereas PD-L2 is limited primarily to dendritic cells (Latchman et al., 2001). Lung tissue expresses PD-L1 and contains activated alveolar macrophages. Therefore, it is likely that anti-PD-1 antibodies remove the inhibitory signals that control tissue proliferation and cytokine production in the lung, whereas anti-PD-L1 antibodies preserve the ligation between PD-1 and PD-L2 (Kong and Flynn, 2014). Moreover, pneumonitis is more commonly observed in patients with NSCLC (Lu et al., 2015) possibly because of difficulties in differentiating pulmonary symptoms and radiographic manifestations caused by treatment from those by disease progression (Lu et al., 2015). The risk factors for drug-related pneumonitis include preexisting ILD (Yamaguchi et al., 2018b) and preexisting pulmonary fibrosis (Jodai et al., 2018; Yamaguchi et al., 2018b). In a previous study in Japanese patients, male gender and smoking history were suggested to be potential risk factors for nivolumab-related pneumonitis (Kato et al., 2017).

Clinical manifestations of pneumonitis range from asymptomatic isolated radiographic abnormalities to a mimic of severe bacterial pneumonia (Sgambato et al., 2016). Onset time of pneumonitis also varies, with the reported range from a few days to over 2 years after treatment initiation (Naidoo et al., 2015; Jodai et al., 2018). Once the patient presents with new pulmonary symptoms, such as cough and shortness of breath, pneumonitis should be suspected (Sgambato et al., 2016). Standard diagnostic algorithms recommend radiologic investigation by chest computed tomography scan. Lung testing, bronchoscopy, and consultations from Infectious Diseases and Pulmonology can be considered in cases of grade ≥2 pneumonitis (Chow, 2013; Sgambato et al., 2016). Differential diagnosis can be a clinical enigma here, and diseases such as infection, early pulmonary edema, congestive heart failure, pulmonary embolus, immune-related tumor inflammation, and tumor progression should all be taken into consideration (Sano et al., 2016; Boyer and Palmer, 2018). Management is guided by clinical symptoms (Topalian et al., 2012; Li et al., 2015). Symptomatic pneumonitis should be monitored daily, and administration of moderate doses (1–2 mg/kg) of prednisone slowly tapered for at least 4 weeks is recommended (Chow, 2013). For patients with severe pneumonitis, a high dose of intravenous steroids (such as 2 mg/kg of methylprednisone) is recommended. Additional immunosuppression with infliximab, mycophenolate mofetil, or cyclophosphamide is reasonable (Postow, 2015). Oxygen and ventilatory support should be applied as appropriate (Chow, 2013). In case the patient’s symptoms are aggressive and severe but differential diagnosis fails between immune-related pneumonitis and immune reactions against tumor cells, management of immune-related pneumonitis should be the priority because immunosuppressant therapy including corticosteroids for irAEs does not affect tumor response (Weber et al., 2015a).

### Dermatologic Toxicity

Dermatologic toxicity is the most common irAE for ICIs (Postow et al., 2015). It occurs in 30–40% of patients treated with anti-PD-1 antibodies, which is comparatively less than the incidence in patients treated with ipilimumab (40–50%) (Belum et al., 2016; Wills et al., 2018). Generally, dermatologic toxicities triggered by anti-PD-1 antibodies are milder and with later onset compared with those triggered by ipilimumab (Palmieri and Carlino, 2018).

The mechanism of PD-1/PD-L1 blockade-induced dermatologic AEs is speculated to be the T-cell homeostasis within the skin, thereby causing self-directed cytotoxic and inflammatory reactions (Okiyama and Katz, 2014). Of note, the combination of nivolumab and radiotherapy might be a risk factor for severe dermatologic AEs, as recently reported in a 77-year-old Japanese patient with advanced melanoma (Tanita et al., 2018) and a 60-year-old Chinese patient with advanced squamous cell lung cancer (Zhao et al., 2018).

Lichenoid reactions, eczema, vitiligo, and pruritus are the most commonly reported dermatologic toxicities after anti-PD-1 monotherapy (Collins et al., 2017). Less common manifestations include lichenoid dermatitis (Joseph et al., 2015), bullous pemphigoid (Carlos et al., 2015), Sweet’s syndrome (Naidoo et al., 2015), and follicular or urticarial dermatitis (Naidoo et al., 2015). Rash and pruritus are two leading AEs of the dermatologic system with PD-1/PD-L1 blockade in Asian trials (**Table 1**). Rash in patients treated with PD-1 inhibitors usually presents as maculopapular lesions on the trunk and extremities within the first few weeks of treatment initiation (Sibaud et al., 2016). A standard workup of dermatological lesions include a comprehensive skin examination, elucidation of prior history of dermatologic conditions, laboratory evaluation of renal and hepatic function panel, and serum levels of tryptase and IgE, as indicated. Skin biopsy should also be considered in selected cases (Wills et al., 2018). Histologic findings might vary among types of immune-related dermatitis. Generally, it often reveals an interface, perivascular and periadnexal lymphocytic dermatitis, with few plasma cells and eosinophils (Naidoo et al., 2015). In a case series of pembrolizumab and nivolumab-induced rash, histopathologic tests revealed perivascular, periadnexal lymphocytic infiltrates with scattered eosinophils (Cramer and Bresalier, 2017).

PD-1/PD-L1 blockade can be continued with caution for grade ≤2 dermatologic AEs. However, consider interrupting it in case the AE does not resolve to grade ≤1 within 1–2 weeks (Haanen et al., 2017). Mild dermatologic AEs can be treated with topical corticosteroids (such as betamethasone or fluocinonide) and oral antipruritic agents (such as antihistamines) (Palmieri and Carlino, 2018; Wills et al., 2018). In case of pruritus involvement, supportive care such as cold compresses and oatmeal baths might alleviate symptoms (Sgambato et al., 2016). Although dermatologic irAEs are usually mild to moderate in severity, rare exfoliative conditions such as Stevens-Johnson syndrome/toxic epidermal necrolysis (Chirasuthat and Chayavichitsilp, 2018) have been observed in Asian patients and can be fatal (Puzanov et al., 2017). In such cases, PD-1/PD-L1 blockade should be permanently discontinued. The patient should be hospitalized immediately. Dermatologic consultation for intravenous corticosteroids, maintenance of fluids, electrolyte monitoring, and appropriate wound care are required (Lu et al., 2015; Zimmermann et al., 2017).

### Endocrinopathy

ICI-related endocrinopathies may affect any axis of the endocrine system, including the pituitary, thyroid, adrenals, and pancreas (Sznol et al., 2017). Specifically, hypophysitis, thyroiditis, hypothyroidism, hyperthyroidism, and Grave’s disease have been seen in ICI therapy (Barroso-Sousa et al., 2018). As previously regarded, hypophysitis occurs mainly with CTLA-4 inhibitors or combinatorial ICIs; dysthyroidism is predominant with PD-1/PD-L1 blockade (Myers, 2018). This is confirmed by the endocrinopathy profile in Asian populations. As observed in early-phase trials in Japan (Hamanishi et al., 2015; Yamazaki et al., 2017), China (Huang et al., 2018), and Asia-Pacific regions (Tahara et al., 2018), hypothyroidism was one of the top 3 most common AEs with anti-PD-1/PD-L1 therapies. In a real-world retrospective study of monotherapy with nivolumab or pembrolizumab in patients with NSCLC in Taiwan, abnormal thyroid function was the most common adverse effect (5/74, 6.5%). Among them, three patients developed hypothyroidism and two developed hyperthyroidism (Lin et al., 2018). In a real-world prospective study in 66 Japanese patients who received nivolumab, destructive thyroiditis was the most frequent endocrine irAE induced, with the onset time from 9 to 60 days (median, 35 days). In addition, patients with positive antithyroglobulin antibodies and/or anti-thyroid peroxidase antibodies at baseline were prone to develop destructive thyroiditis after initiation of nivolumab (Kobayashi et al., 2018). In PD-1/PD-L1 blockade-related irAEs, rare observed endocrinopathies include primary adrenal insufficiency, insulin-dependent diabetes mellitus (type I), hypercalcemia, and hypoparathyroidism (Wills et al., 2018). Specifically, fulminant type 1 diabetes has recently been discovered as an important subtype, especially in East Asia. It accounts for approximately 20% of acute-onset type 1 diabetes in Japan (Imagawa et al., 2000; Matsuura et al., 2018).

The symptoms of immune-related endocrinopathies are usually nonspecific, such as fatigue, headache, and nausea, which are especially common for cancer patients (Geukes Foppen et al., 2017; Sosa et al., 2018). Therefore, laboratory monitoring of endocrine function is a fundamental method in the diagnosis. Imaging tests, such as magnetic resonance imaging, are indicated in selected cases (Postow, 2015). A characteristic of immune-related endocrinopathies is that the development of disease is typically irreversible (Myers, 2018). Fortunately, endocrinopathies could be easily managed with hormone supplementation or replacement, such as levothyroxine for a hypothyroid status (Postow et al., 2015). For symptomatic hypophysitis, adrenal crisis, or severe thyrotoxicosis, such as thyroid storm, short-term high-dose corticosteroids are required (Illouz et al., 2017; Brahmer et al., 2018). As majority of immune-related endocrinopathies can be treated successfully with hormone replacement, anti-PD-1/PD-L1 therapy is not usually discontinued under the premise of close monitoring of treatment response and endocrine functions (Naidoo et al., 2015; Sgambato et al., 2016).

### Gastrointestinal Toxicity

Diarrhea and colitis account for the most gastrointestinal toxicities with PD-1/PD-L1 blockade across populations (Kudo et al., 2017). Upon the diagnosis of immune-related diarrhea, infection with *Clostridium difficile* or other pathogens shall be ruled out. For mild diarrhea, oral hydration, electrolyte substitution, and antimotility agents (such as loperamide) can be adopted with close monitoring (Haanen et al., 2017; Puzanov et al., 2017; Brahmer et al., 2018). In case antimotility agents are not appropriate, consider low-dose systemic corticosteroids or local budesonide (Haanen et al., 2017; Brahmer et al., 2018). In clinically serious cases, anti-PD-1/PD-L1 agents should be discontinued, and the patients can be hospitalized for intravenous corticosteroids (such as prednisone 1 mg/kg daily). If no response to corticosteroids is observed, or the condition relapses after corticosteroids, additional immunosuppression with anti-tumor necrosis factor agents (such as a single dose of infliximab 5 mg/kg) can be considered (Lu et al., 2015; Haanen et al., 2017; Prieux-Klotz et al., 2017; Brahmer et al., 2018).

### Renal Toxicity

Renal AEs related to PD-1/PD-L1 blockade are comparatively less common in Asian populations. In a multicenter phase 2 study of nivolumab in Japanese patients with advanced or recurrent NSCLC, renal toxicities were reported in 5.3% (4/76) of patients (Nishio et al., 2017). In other trials of anti-PD-1/PD-L1 antibodies in Asians, treatment-related renal AEs were scarcely seen. Very rare cases reported in Asian patients also involved acute granulomatous tubulointerstitial nephritis (Nakatani et al., 2018) and minimal change in the disease. The patients are usually asymptomatic despite an elevated creatinine identified from routine laboratory tests. Therefore, frequent monitoring of renal function indexes is recommended throughout the entire process of anti-PD-1/PD-L1 treatment. If immune-related nephropathy is suspected, renal biopsy might be considered for definite diagnosis unless contraindicated (Boussiotis, 2016). For severe immune-related kidney injury, potential nephrotoxic agents shall be avoided, and corticosteroids and discontinuation of anti-PD-1/PD-L1 therapy are recommended (Boussiotis, 2016). In case of renal function recovery, anti-PD-1/PD-L1 therapy can be reintroduced with caution (Nakatani et al., 2018).

### Ocular Toxicity

Ocular irAEs occur in <1% of patients receiving ICIs (Antoun et al., 2016), and common ocular manifestations include episcleritis, conjunctivitis, and uveitis (Antoun et al., 2016). In clinical trials in Asians, uveitis was seen in 3% (1/38) in a phase 1b study (KEYNOTE-025) of pembrolizumab in Japanese patients with previously treated PD-L1-positive advanced NSCLC (Nishio et al., 2019). In a phase 1 study of camrelizumab in Chinese patients with advanced solid tumors, conjunctivitis was observed in one of 12 patients who received intravenous camrelizumab at 60 mg but not at higher dosage levels (4-week interval after first dose followed by a 2-week schedule) (Mo et al., 2018). Moreover, in a multicenter phase 2 study of nivolumab in Japanese patients with relapsed or refractory classical Hodgkin lymphoma, cataract was seen in 11.8% (2/17) of patients (Maruyama et al., 2017). For any visual complaints during anti-PD-1/PD-L1 therapy, ophthalmologic assessment including dilated fundoscopy and slit lamp examination should be performed promptly (Puzanov et al., 2017). Mild ocular irAEs may resolve spontaneously or can be treated with topical corticosteroids, whereas oral or systemic corticosteroids are indicated for more severe cases (Kumar et al., 2017). An ocular condition that calls for extra attention is immune-related uveitis, which is rare but may result in irreversible visual loss if not properly managed (Wang et al., 2019). A case report presented a 64-year-old Chinese female who developed grade 4 panuveitis with bilateral serous retinal detachment after treatment with nivolumab for metastatic renal cell carcinoma (Wang et al., 2019). In that patient, pulsed intravenous methylprednisolone and oral prednisone improved visual acuity and retinal detachment. However, uveitis relapsed 2 weeks after reinitiation of nivolumab. In the end, intravitreal injection of dexamethasone implant, but not the periorbital injection of steroid or the steroid eye drops, was effective to control the posterior uveitis and serous retinal detachment (Wang et al., 2019).

### Immune-Related Adverse Events in Other Organ Systems

Theoretically, any organ system of the body can be affected with irAEs. In Asian cancer patients treated with anti-PD-1/PD-L1 antibodies, other sporadically reported AEs include neuroskeletomuscular toxicities such as neuromyelitis optica spectrum disorder (Narumi et al., 2018), akathisia (Reyes et al., 2016), and myasthenia gravis (Mitsune et al., 2018); cardiotoxicities such as acute coronary syndrome (Wang et al., 2017), fulminant myocarditis (Yamaguchi et al., 2018a), sick sinus syndrome (Hsu et al., 2018a), and rhabdomyolysis (Chen et al., 2018); coagulopathies such as acute thrombosis (Kunimasa et al., 2018) and Trousseau’s syndrome (Horio et al., 2018); and rheumatologic toxicities such as inflammatory arthritis (Inamo et al., 2018).

## Management of Patients with Preexisting Infectious Conditions

There have been concerns whether PD-1/PD-L1 blockade exacerbates preexisting conditions that were well maintained without turbulence in immune homeostasis (Mitsune et al., 2018). In Asian patients with cancers, a particular condition that needs extra consideration is the preexisting chronic infection of certain viruses. Hepatitis B virus (HBV) infection is a major public health problem globally. As reported by the Polaris Observatory Collaborators, Asian (Central, East, and Southeast) and Sub-Saharan Africa are two major regions with the highest prevalence of HBV (Collaborators, 2018). In China, despite the drop of incidence and mortality of HBV infection, thanks to a national program for HBV immunization, China certainly confronts the largest number of patients with HBV infection in the world for the size of the population. Chronic HBV infection remains a prominent cause of liver cancer in China (Xiao et al., 2019). Immune dysregulation modulates the entire process of HBV-associated liver diseases from hepatitis to HBV-related hepatocellular carcinoma (HCC) (Li et al., 2016; Cho et al., 2017; Trehanpati and Vyas, 2017). In chronic viral hepatitis, the extended upregulation of PD-1 and CTLA-4 is associated with T-cell exhaustion and persistent viral infection, suggesting that the expressions of immune inhibitory factors are positively associated with the chronicity of viral disease (Shun et al., 2019). Currently, immunotherapy that inhibits immune checkpoint pathway is being tested as a new approach for the cure of HBV (Shire, 2017). And in HBV-related HCC or HBV carriers with cancer, ICIs might benefit both virus relapse and tumor progression theoretically (Shun et al., 2019). In a study of HCC patients treated with tremelimumab (a CTLA-4 inhibitor) in combination with ablation, five patients with hepatitis B were enrolled. In these patients, quantitative hepatitis B antigen was found to decrease over time in all patients, and no viral reactivation was seen (Duffy et al., 2017). However, in a retrospective study of ICI in Taiwan, 12 patients were hepatitis B carriers. Among them, one patient contracted hepatitis. Later, the patient was suspected of hepatitis B recurrence and resistance to entecavir. Hence, the original drug was switched to tenofovir (Hsu et al., 2018b).

Here, a similar situation involves the infection of tuberculosis (TB). In a recent case report, pulmonary TB of a 65-year-old Chinese female was activated after administration of pembrolizumab for metastatic melanoma. Immunotherapy was suspended, and anti-TB drugs were administered, followed by pembrolizumab (He et al., 2018).

As the contrainteraction among viruses, cancers, and PD-1/PD-L1 inhibitors remains unclear, physicians shall bear in mind the reactivation of latent infection and opportunistic infection as potential AEs when managing cancer patients with PD-1/PD-L1 blockade, especially for patients from endemic areas (Lee et al., 2016; Reungwetwattana and Adjei, 2016). At present, carriers of viruses such as HBV, TB, and HIV were routinely excluded from clinical trials. Therefore, there is a lack of information for anti-PD-1/PD-L1 treatment in patients with existing infectious conditions. With limited regulations for clinical practice, screening for major viruses such as HIV, TB, and HBV (especially for patients with HCC) according to the prevalence before initiation of PD-1/PD-L1 blockade is encouraged (Reungwetwattana and Adjei, 2016; He et al., 2018).

## Limitations

Limitations of the statistical analyses performed in this review shall be addressed. First, the incidence of AEs included for statistical analyses comes from studies on different anti-PD-1/PD-L1 agents. The dosages and the frequencies of administration can be inconsistent among studies with the same therapeutic agent. Moreover, the studies were performed in patients with different types of cancers, and significant heterogeneity across studies may exist. Second, the standard definitions of AE, irAE, or TRAE were yet to be established. Thus, the definitions adopted in the studies might be inconsistent or even subjective to the investigators. Therefore, the results of statistical analysis in this review shall be interpreted with caution. While it depicted a generally different profile of AEs between Western/international patients and Asian patients treated with PD-1/PD-L1 blockade, the exact prevalence of certain AEs shall be determined by clinical studies with large sample sizes.

## Current Challenges and Future Perspectives

While PD-1/PD-L1 blockade is revolutionizing the treatment in oncology, it leads to a new spectrum of AEs. In the field of the management of AEs with immunotherapy, the balance between control of irAEs and maintenance of antitumor effect has been a recent research focus. Because PD-1/PD-L1 blockade works by enhancing antitumor immunity, it has been wondered whether treatment of irAEs by immunosuppression would impair the antitumor efficacy of PD-1/PD-L1 blockade. In several retrospective studies, irAEs were associated with favorable clinical outcomes including tumor response (Ishihara et al., 2017; Kim et al., 2018) and survival (Teulings et al., 2015; Yamazaki et al., 2017). Nevertheless, the results remain controversial (Weber et al., 2017), and it cannot be ruled out that nonresponder patients discontinued PD-1/PD-L1 blockade before the onset of irAEs (Yamazaki et al., 2017). Therefore, studies exploring the exact relationships between treatment of irAEs and clinical outcomes are needed to select the right time for immunosuppressive intervention of AEs and to obtain a balance between minimal toxicity and optimal antitumor efficacy. In addition, there have been few studies focusing on the management strategies for AEs with PD-1/PD-L1 blockade. Current management is mainly based on the guidelines developed for other ICIs such as CTLA-4 inhibitors, and lots of ambiguities remain. In the future, experimental studies and clinical studies with large sample sizes may further elucidate the mechanism and reveal the characteristics of AEs in patients treated with PD-1/PD-L1 blockade. Potential research interests might include prophylaxis of AEs and individualized dosing regimens of PD-1/PD-L1 blockade.

By now, only a small number of clinical trials in Asian populations have reported outcomes, and included patients were mainly from Japan, China, and South Korea. The profile of AEs in Asians needs to be further depicted in the future. As concluded in the current review, the characteristics of AEs in Asian populations might be different from those in Western patients with cancers. Along with the growing body of information of AEs with PD-1/PD-L1 blockade, tools of precision medicine shall be applied to determine the optimal management strategy of AEs in cancer patients of different races or other characteristics. Moreover, guidelines that adapt to types of AEs in certain populations shall be refined and updated pertinently.

## Author Contributions

JY and HS conceptualized this review. JY wrote and edited the manuscript. JY and XH created the figures. QL, JJ, and HS revised and edited the manuscript.

## Funding

This work was supported by (1) National Key Research and Development Program of China (No. 2016YFC0906000 [2016YFC​0906003]); (2) National Natural Science Foundation of China (No. 81773752); (3) Key Program of the Science and Technology Bureau of Sichuan (No. 2017SZ00005); (4) Post-Doctor Research Project, West China Hospital, Sichuan University (No. 2019HXBH046). HS is supported by the grant from “The Recruitment Program of Global Young Experts” (known as “the Thousand Young Talents Plan”).

## Conflict of Interest Statement

The authors declare that the research was conducted in the absence of any commercial or financial relationships that could be construed as a potential conflict of interest.
