# Supplementary material for: Management of Adverse Events in Cancer Patients Treated With PD-1/PD-L1 Blockade: Focus on Asian Populations
Source: Front Pharmacol. 2019 Jul 2;10:726. doi: 10.3389/fphar.2019.00726 (PMC6614522; doi:10.3389/fphar.2019.00726)
Supplement: Supplementary file 1 [file Table_1.docx]

**Management of adverse events in cancer patients treated with PD-1/PD-L1 blockade: Focus on Asian population**

# Supplementary Table 1. Characteristics of clinical trials on PD-1/PD-L1 blockades in patients with malignancies.

| **Population** | **Agent** | **Trial name** | **Trial number** | **Phase** | **Sample size** | **Type of cancer** | **Reference** |
| --- | --- | --- | --- | --- | --- | --- | --- |
| Asian | Atezolizumab |  | JapicCTI-132208 | 1 | 6 | Solid tumors | (Mizugaki et al., 2016) |
| Asian | Avelumab |  | NCT01943461 | 1 | 57 | Solid tumors | (Doi et al., 2018) |
| Asian | Camrelizumab |  | NCT02721589 | 1 | 93 | Nasopharyngeal carcinoma | (Fang et al., 2018) |
| Asian | Camrelizumab |  | NCT02742935 | 1 | 30 | Gastric or gastroesophageal junction cancer | (Huang et al., 2019) |
| Asian | Camrelizumab |  | NCT02742935 | 1 | 30 | Esophageal carcinoma | (Huang et al., 2018) |
| Asian | Camrelizumab |  | NCT02742935 | 1 | 36 | Solid tumors | (Mo et al., 2018) |
| Asian | Nivolumab |  | UMIN000005714 | 2 | 20 | Ovarian cancer | (Hamanishi et al., 2015) |
| Asian | Nivolumab |  | NCT02267343 | 3 | 330 | Gastric or gastroesophageal junction cancer | (Kang et al., 2017) |
| Asian | Nivolumab |  | JapicCTI-142422 | 2 | 65 | Esophageal carcinoma | (Kudo et al., 2017) |
| Asian | Nivolumab |  | JapicCTI-142755 | 2 | 17 | Hodgkin lymphoma | (Maruyama et al., 2017) |
| Asian | Nivolumab |  | JapicCTI-132073 | 2 | 76 | Non-small-cell lung carcinoma | (Nishio et al., 2017) |
| Asian | Nivolumab |  | JapicCTI-142533 | 2 | 24 | Melanoma | (Yamazaki et al., 2017) |
| Asian | Pembrolizumab |  | NCT02007070 | 1b | 38 | Non-small-cell lung carcinoma | (Nishio et al., 2019) |
| Asian | Pembrolizumab |  | NCT01840579 | 1 | 10 | Solid tumors | (Shimizu et al., 2016) |
| Asian | Pembrolizumab |  | NCT01848834 | 1b | 26 | Head and neck squamous cell carcinoma | (Tahara et al., 2018) |
| Western/international | Atezolizumab |  | NCT01375842 | 1 | 32 | Head and neck cancer | (Colevas et al., 2018) |
| Western/international | Atezolizumab |  | NCT01375842 | 1 | 116 | Triple-negative breast cancer | (Emens et al., 2019) |
| Western/international | Atezolizumab | POPLAR | NCT01903993 | 2 | 142 | Non-small-cell lung carcinoma | (Fehrenbacher et al., 2016) |
| Western/international | Atezolizumab | PCD4989g | NCT01375842 | 1a | 16 | Glioblastoma | (Lukas et al., 2018) |
| Western/international | Atezolizumab | BIRCH | NCT02031458 | 2 | 659 | Non-small-cell lung carcinoma | (Peters et al., 2017) |
| Western/international | Atezolizumab | IMvigor211 | NCT02302807 | 3 | 459 | Urothelial carcinoma | (Powles et al., 2018) |
| Western/international | Atezolizumab | IFCT-1603 | NCT03059667 | 2 | 48 | Non-small-cell lung carcinoma | (Pujol et al., 2019) |
| Western/international | Atezolizumab | OAK | NCT02008227 | 3 | 609 | Non-small-cell lung carcinoma | (Rittmeyer et al., 2017) |
| Western/international | Atezolizumab |  | NCT02108652 | 2 | 310 | Urothelial carcinoma | (Rosenberg et al., 2016) |
| Western/international | Avelumab | JAVELIN Lung 200 | NCT02395172 | 3 | 393 | Non-small-cell lung carcinoma | (Barlesi et al., 2018) |
| Western/international | Avelumab | JAVELIN Solid Tumor | NCT01772004 | 1b | 150 | Gastric or gastroesophageal junction cancer | (Chung et al., 2019) |
| Western/international | Avelumab | JAVELIN Solid Tumor | NCT01772004 | 1b | 168 | Breast cancer | (Dirix et al., 2018) |
| Western/international | Avelumab | JAVELIN Solid Tumor | NCT01772004 | 1 | 184 | Non-small-cell lung carcinoma | (Gulley et al., 2017) |
| Western/international | Avelumab | JAVELIN Solid Tumor | NCT01772004 | 1b | 53 | Mesothelioma | (Hassan et al., 2019) |
| Western/international | Avelumab | JAVELIN Solid Tumor | NCT01772004 | 1a | 53 | Solid tumours | (Heery et al., 2017) |
| Western/international | Avelumab |  | NCT02155647 | 2 | 88 | Merkel cell carcinoma | (Kaufman et al., 2016) |
| Western/international | Avelumab | JAVELIN Solid Tumor | NCT01772004 | 1b | 51 | Melanoma | (Keilholz et al., 2018) |
| Western/international | Avelumab | JAVELIN Solid Tumor | NCT01772004 | 1b | 25 | Adrenocortical carcinoma | (Le Tourneau et al., 2018) |
| Western/international | Avelumab | JAVELIN Solid Tumor | NCT01772004 | 1 | 249 | Urothelial carcinoma | (Patel et al., 2018) |
| Western/international | Durvalumab | ATLANTIC | NCT02087423 | 2 | 444 | Non-small-cell lung carcinoma | (Garassino et al., 2018) |
| Western/international | Durvalumab |  | NCT01693562 | 1/2 | 970 | Urothelial carcinoma | (Powles et al., 2017) |
| Western/international | Durvalumab | CONDOR | NCT02319044 | 2 | 65 | Head and neck squamous cell carcinoma | (Siu et al., 2019) |
| Western/international | Nivolumab |  | NCT02428192 | 2 | 12 | Leiomyosarcoma of the uterus | (Ben-Ami et al., 2017) |
| Western/international | Nivolumab |  | NCT00729664 | 1 | 207 | Advanced cancers | (Brahmer et al., 2012) |
| Western/international | Nivolumab | CheckMate 026 | NCT02041533 | 3 | 267 | Non-small-cell lung carcinoma | (Carbone et al., 2017) |
| Western/international | Nivolumab | Alliance A091401 | NCT02500797 | 2 | 42 | Metastatic sarcoma | (D’Angelo et al., 2018) |
| Western/international | Nivolumab | CheckMate 040 | NCT01658878 | 1/2 | 48 | Hepatocellular carcinoma | (El-Khoueiry et al., 2017) |
| Western/international | Nivolumab | CheckMate 141 | NCT0210563 | 3 | 236 | Head and neck squamous cell carcinoma | (Ferris et al., 2016) |
| Western/international | Nivolumab | Checkmate 012 | NCT01454102 | 1 | 52 | Non-small-cell lung carcinoma | (Gettinger et al., 2016) |
| Western/international | Nivolumab | CheckMate 067 | NCT01844505 | 3 | 313 | Melanoma | (Hodi et al., 2018) |
| Western/international | Nivolumab | NCI9673 | NCT02314169 | 2 | 37 | Anal cancer | (Morris et al., 2017) |
| Western/international | Nivolumab | CheckMate 142 | NCT02060188 | 2 | 74 | Colorectal cancer | (Overman et al., 2017) |
| Western/international | Nivolumab | NivoMes | NCT02497508 | 2 | 34 | Malignant mesothelioma | (Quispel-Janssen et al., 2018) |
| Western/international | Nivolumab | CheckMate 063 | NCT01721759 | 2 | 117 | Non-small-cell lung carcinoma | (Rizvi et al., 2015) |
| Western/international | Nivolumab | IFCT-1501 MAPS2 | NCT02716272 | 2 | 63 | Malignant pleural mesothelioma | (Scherpereel et al., 2019) |
| Western/international | Nivolumab | CheckMate 032 | NCT01928394 | 1/2 | 78 | Urothelial carcinoma | (Sharma et al., 2016) |
| Western/international | Nivolumab | CheckMate 275 | NCT02387996 | 2 | 270 | Urothelial carcinoma | (Sharma et al., 2017) |
| Western/international | Nivolumab |  | NCT00730639 | 1 | 107 | Melanoma | (Topalian et al., 2014) |
| Western/international | Nivolumab | CheckMate 037 | NCT01721746 | 3 | 272 | Melanoma | (Weber et al., 2015) |
| Western/international | Nivolumab |  | NCT02181738 | 2 | 80 | Classical Hodgkin lymphoma | (Younes et al., 2016) |
| Western/international | Pembrolizumab | KEYNOTE-086 | NCT02447003 | 2 | 170 | Triple-negative breast cancer | (Adams et al., 2018) |
| Western/international | Pembrolizumab | GU14-206 | NCT02499952 | 2 | 12 | Germ-cell tumors | (Adra et al., 2017) |
| Western/international | Pembrolizumab | KEYNOTE-028 | NCT02054806 | 1b | 25 | Malignant pleural mesothelioma | (Alley et al., 2017) |
| Western/international | Pembrolizumab | KEYNOTE-052 | NCT02335424 | 2 | 370 | Urothelial cancer | (Balar et al., 2017) |
| Western/international | Pembrolizumab | KEYNOTE-045 | NCT02256436 | 3 | 266 | Urothelial carcinoma | (Bellmunt et al., 2017) |
| Western/international | Pembrolizumab | KEYNOTE-012 | NCT01848834 | 1b | 132 | Head and neck squamous cell carcinoma | (Chow et al., 2016) |
| Western/international | Pembrolizumab | KEYNOTE-028 | NCT02054806 | 1b | 26 | Salivary gland carcinoma | (Cohen et al., 2018) |
| Western/international | Pembrolizumab |  | NCT02362594 | 3 | 509 | Melanoma | (Eggermont et al., 2018) |
| Western/international | Pembrolizumab | KEYNOTE-059 | NCT02335411 | 2 | 259 | Gastric or gastroesophageal junction cancer | (Fuchs et al., 2018) |
| Western/international | Pembrolizumab |  | NCT02085070 | 2 | 36 | Melanoma or Non-small-cell lung carcinoma | (Goldberg et al., 2016) |
| Western/international | Pembrolizumab | KEYNOTE-002 | NCT01704287 | 2 | 180 | Melanoma | (Hamid et al., 2017) |
| Western/international | Pembrolizumab | KEYNOTE-028 | NCT02054806 | 1b | 23 | Prostate adenocarcinoma | (Hansen et al., 2018) |
| Western/international | Pembrolizumab | KEYNOTE-010 | NCT01905657 | 2/3 | 682 | Non-small-cell lung carcinoma | (Herbst et al., 2016) |
| Western/international | Pembrolizumab | KEYNOTE-028 | NCT02054806 | 1b | 27 | Nasopharyngeal carcinoma | (Hsu et al., 2017) |
| Western/international | Pembrolizumab | KEYNOTE-001 | NCT01295827 | 1 | 101 | Non-small-cell lung carcinoma | (Hui et al., 2017) |
| Western/international | Pembrolizumab |  | NCT02879994 | 2 | 11 | Non-small-cell lung carcinoma | (Lisberg et al., 2018) |
| Western/international | Pembrolizumab | KEYNOTE-028 | NCT02054806 | 1b | 22 | Papillary or follicular thyroid cancer | (Mehnert et al., 2019) |
| Western/international | Pembrolizumab | KEYNOTE-012 | NCT01848834 | 1b | 39 | Gastric cancer | (Muro et al., 2016) |
| Western/international | Pembrolizumab | KEYNOTE-012 | NCT01848834 | 1b | 32 | Triple-negative breast cancer | (Nanda et al., 2016) |
| Western/international | Pembrolizumab | KEYNOTE-028 | NCT02054806 | 1b | 23 | Colorectal carcinoma | (O’Neil et al., 2017) |
| Western/international | Pembrolizumab | KEYNOTE-028 | NCT02054806 | 1b | 25 | Carcinoma of the anal canal | (Ott et al., 2017) |
| Western/international | Pembrolizumab | KEYNOTE-012 | NCT01848834 | 1b | 33 | Urothelial cancer | (Plimack et al., 2017) |
| Western/international | Pembrolizumab | KEYNOTE-024 | NCT02142738 | 3 | 154 | Non-small-cell lung carcinoma | (Reck et al., 2016) |
| Western/international | Pembrolizumab |  | NCT01295827 | 1 | 173 | Melanoma | (Robert et al., 2014) |
| Western/international | Pembrolizumab | KEYNOTE-006 | NCT01866319 | 3 | 555 | Melanoma | (Schachter et al., 2017) |
| Western/international | Pembrolizumab | KEYNOTE-180 | NCT02559687 | 2 | 121 | Adenocarcinoma or squamous cell carcinoma of the esophagus | (Shah et al., 2018) |
| Western/international | Pembrolizumab | KEYNOTE-061 | NCT02370498 | 3 | 294 | Gastric or gastroesophageal junction cancer | (Shitara et al., 2018) |
| Western/international | Pembrolizumab | SARC028 | NCT02301039 | 2 | 84 | Soft-tissue sarcoma and bone sarcoma | (Tawbi et al., 2017) |
| Western/international | Pembrolizumab | KEYNOTE-028 | NCT02054806 | 1b | 26 | Ovarian cancer | (Varga et al., 2019) |
| Western/international | Pembrolizumab | KEYNOTE-013 | NCT01953692 | 1 | 18 | Primary mediastinal large B-cell lymphoma | (Zinzani et al., 2017) |

**Reference**

Adams, S., Schmid, P., Rugo, H., Winer, E., Loirat, D., Awada, A., et al. (2018). Pembrolizumab monotherapy for previously treated metastatic triple-negative breast cancer: cohort A of the phase II KEYNOTE-086 study. *Ann Oncol* 30(3)**,** 397-404.

Adra, N., Einhorn, L.H., Althouse, S.K., Ammakkanavar, N.R., Musapatika, D., Albany, C., et al. (2017). Phase II trial of pembrolizumab in patients with platinum refractory germ-cell tumors: a Hoosier Cancer Research Network Study GU14-206. *Ann Oncol* 29(1)**,** 209-214.

Alley, E.W., Lopez, J., Santoro, A., Morosky, A., Saraf, S., Piperdi, B., et al. (2017). Clinical safety and activity of pembrolizumab in patients with malignant pleural mesothelioma (KEYNOTE-028): preliminary results from a non-randomised, open-label, phase 1b trial. *Lancet Oncol* 18(5)**,** 623-630.

Balar, A.V., Castellano, D., O'Donnell, P.H., Grivas, P., Vuky, J., Powles, T., et al. (2017). First-line pembrolizumab in cisplatin-ineligible patients with locally advanced and unresectable or metastatic urothelial cancer (KEYNOTE-052): a multicentre, single-arm, phase 2 study. *Lancet Oncol* 18(11)**,** 1483-1492.

Barlesi, F., Vansteenkiste, J., Spigel, D., Ishii, H., Garassino, M., de Marinis, F., et al. (2018). Avelumab versus docetaxel in patients with platinum-treated advanced non-small-cell lung cancer (JAVELIN Lung 200): an open-label, randomised, phase 3 study. *Lancet Oncol* 19(11)**,** 1468-1479.

Bellmunt, J., De Wit, R., Vaughn, D.J., Fradet, Y., Lee, J.-L., Fong, L., et al. (2017). Pembrolizumab as second-line therapy for advanced urothelial carcinoma. *N Engl J Med* 376(11)**,** 1015-1026.

Ben-Ami, E., Barysauskas, C.M., Solomon, S., Tahlil, K., Malley, R., Hohos, M., et al. (2017). Immunotherapy with single agent nivolumab for advanced leiomyosarcoma of the uterus: Results of a phase 2 study. *Cancer* 123(17)**,** 3285-3290.

Brahmer, J.R., Tykodi, S.S., Chow, L.Q., Hwu, W.-J., Topalian, S.L., Hwu, P., et al. (2012). Safety and activity of anti–PD-L1 antibody in patients with advanced cancer. *N Engl J Med* 366(26)**,** 2455-2465.

Carbone, D.P., Reck, M., Paz-Ares, L., Creelan, B., Horn, L., Steins, M., et al. (2017). First-line nivolumab in stage IV or recurrent non–small-cell lung cancer. *N Engl J Med* 376(25)**,** 2415-2426.

Chow, L.Q., Haddad, R., Gupta, S., Mahipal, A., Mehra, R., Tahara, M., et al. (2016). Antitumor activity of pembrolizumab in biomarker-unselected patients with recurrent and/or metastatic head and neck squamous cell carcinoma: results from the phase Ib KEYNOTE-012 expansion cohort. *J Clin Oncol* 34(32)**,** 3838-3845.

Chung, H.C., Arkenau, H.-T., Lee, J., Rha, S.Y., Oh, D.-Y., Wyrwicz, L., et al. (2019). Avelumab (anti–PD-L1) as first-line switch-maintenance or second-line therapy in patients with advanced gastric or gastroesophageal junction cancer: phase 1b results from the JAVELIN Solid Tumor trial. *J Immunother Cancer* 7(1)**,** 30.

Cohen, R.B., Delord, J.-P., Doi, T., Piha-Paul, S.A., Liu, S.V., Gilbert, J., et al. (2018). Pembrolizumab for the treatment of advanced salivary gland carcinoma: findings of the phase 1b KEYNOTE-028 study. *Am J Clin Oncol* 41(11)**,** 1083.

Colevas, A., Bahleda, R., Braiteh, F., Balmanoukian, A., Brana, I., Chau, N., et al. (2018). Safety and clinical activity of atezolizumab in head and neck cancer: results from a phase I trial. *Ann Oncol* 29(11)**,** 2247-2253.

D’Angelo, S.P., Russell, J., Lebbé, C., Chmielowski, B., Gambichler, T., Grob, J.-J., et al. (2018). Efficacy and safety of first-line avelumab treatment in patients with stage IV metastatic Merkel cell carcinoma: a preplanned interim analysis of a clinical trial. *JAMA Oncol* 4(9)**,** e180077-e180077.

Dirix, L.Y., Takacs, I., Jerusalem, G., Nikolinakos, P., Arkenau, H.-T., Forero-Torres, A., et al. (2018). Avelumab, an anti-PD-L1 antibody, in patients with locally advanced or metastatic breast cancer: a phase 1b JAVELIN Solid Tumor study. *Breast Cancer Res Treat* 167(3)**,** 671-686.

Doi, T., Iwasa, S., Muro, K., Satoh, T., Hironaka, S., Esaki, T., et al. (2018). Phase 1 trial of avelumab (anti-PD-L1) in Japanese patients with advanced solid tumors, including dose expansion in patients with gastric or gastroesophageal junction cancer: the JAVELIN Solid Tumor JPN trial. *Gastric Cancer***,** 1-11.

Eggermont, A.M., Blank, C.U., Mandala, M., Long, G.V., Atkinson, V., Dalle, S., et al. (2018). Adjuvant pembrolizumab versus placebo in resected stage III melanoma. *N Engl J Med* 378(19)**,** 1789-1801.

El-Khoueiry, A.B., Sangro, B., Yau, T., Crocenzi, T.S., Kudo, M., Hsu, C., et al. (2017). Nivolumab in patients with advanced hepatocellular carcinoma (CheckMate 040): an open-label, non-comparative, phase 1/2 dose escalation and expansion trial. *Lancet* 389(10088)**,** 2492-2502.

Emens, L.A., Cruz, C., Eder, J.P., Braiteh, F., Chung, C., Tolaney, S.M., et al. (2019). Long-term clinical outcomes and biomarker analyses of atezolizumab therapy for patients with metastatic triple-negative breast cancer: a phase 1 study. *JAMA Oncol* 5(1)**,** 74-82.

Fang, W., Yang, Y., Ma, Y., Hong, S., Lin, L., He, X., et al. (2018). Camrelizumab (SHR-1210) alone or in combination with gemcitabine plus cisplatin for nasopharyngeal carcinoma: results from two single-arm, phase 1 trials. *Lancet Oncol* 19(10)**,** 1338-1350.

Fehrenbacher, L., Spira, A., Ballinger, M., Kowanetz, M., Vansteenkiste, J., Mazieres, J., et al. (2016). Atezolizumab versus docetaxel for patients with previously treated non-small-cell lung cancer (POPLAR): a multicentre, open-label, phase 2 randomised controlled trial. *Lancet* 387(10030)**,** 1837-1846.

Ferris, R.L., Blumenschein Jr, G., Fayette, J., Guigay, J., Colevas, A.D., Licitra, L., et al. (2016). Nivolumab for recurrent squamous-cell carcinoma of the head and neck. *N Engl J Med* 375(19)**,** 1856-1867.

Fuchs, C.S., Doi, T., Jang, R.W., Muro, K., Satoh, T., Machado, M., et al. (2018). Safety and efficacy of pembrolizumab monotherapy in patients with previously treated advanced gastric and gastroesophageal junction cancer: phase 2 clinical KEYNOTE-059 trial. *JAMA Oncol* 4(5)**,** e180013-e180013.

Garassino, M.C., Cho, B.-C., Kim, J.-H., Mazières, J., Vansteenkiste, J., Lena, H., et al. (2018). Durvalumab as third-line or later treatment for advanced non-small-cell lung cancer (ATLANTIC): an open-label, single-arm, phase 2 study. *Lancet Oncol* 19(4)**,** 521-536.

Gettinger, S.N., Rizvi, N.A., Chow, L.Q., Borghaei, H., Brahmer, J.R., Ready, N., et al. (2016). Nivolumab monotherapy for first-line treatment of advanced non–small-cell lung cancer. *J Clin Oncol* 34(25)**,** 2980.

Goldberg, S.B., Gettinger, S.N., Mahajan, A., Chiang, A.C., Herbst, R.S., Sznol, M., et al. (2016). Pembrolizumab for patients with melanoma or non-small-cell lung cancer and untreated brain metastases: early analysis of a non-randomised, open-label, phase 2 trial. *Lancet Oncol* 17(7)**,** 976-983.

Gulley, J.L., Rajan, A., Spigel, D.R., Iannotti, N., Chandler, J., Wong, D.J., et al. (2017). Avelumab for patients with previously treated metastatic or recurrent non-small-cell lung cancer (JAVELIN Solid Tumor): dose-expansion cohort of a multicentre, open-label, phase 1b trial. *Lancet Oncol* 18(5)**,** 599-610.

Hamanishi, J., Mandai, M., Ikeda, T., Minami, M., Kawaguchi, A., Murayama, T., et al. (2015). Safety and antitumor activity of anti–PD-1 antibody, nivolumab, in patients with platinum-resistant ovarian cancer. *J Clin Oncol* 33(34)**,** 4015-4022.

Hamid, O., Puzanov, I., Dummer, R., Schachter, J., Daud, A., Schadendorf, D., et al. (2017). Final analysis of a randomised trial comparing pembrolizumab versus investigator-choice chemotherapy for ipilimumab-refractory advanced melanoma. *Eur J Cancer* 86**,** 37-45.

Hansen, A., Massard, C., Ott, P., Haas, N., Lopez, J., Ejadi, S., et al. (2018). Pembrolizumab for advanced prostate adenocarcinoma: findings of the KEYNOTE-028 study. *Ann Oncol* 29(8)**,** 1807-1813.

Hassan, R., Thomas, A., Nemunaitis, J.J., Patel, M.R., Bennouna, J., Chen, F.L., et al. (2019). Efficacy and safety of avelumab treatment in patients with advanced unresectable mesothelioma: phase 1b results from the JAVELIN solid tumor trial. *JAMA Oncol* 5(3)**,** 351-357.

Heery, C.R., O'Sullivan-Coyne, G., Madan, R.A., Cordes, L., Rajan, A., Rauckhorst, M., et al. (2017). Avelumab for metastatic or locally advanced previously treated solid tumours (JAVELIN Solid Tumor): a phase 1a, multicohort, dose-escalation trial. *Lancet Oncol* 18(5)**,** 587-598.

Herbst, R.S., Baas, P., Kim, D.-W., Felip, E., Pérez-Gracia, J.L., Han, J.-Y., et al. (2016). Pembrolizumab versus docetaxel for previously treated, PD-L1-positive, advanced non-small-cell lung cancer (KEYNOTE-010): a randomised controlled trial. *Lancet* 387(10027)**,** 1540-1550.

Hodi, F.S., Chiarion-Sileni, V., Gonzalez, R., Grob, J.-J., Rutkowski, P., Cowey, C.L., et al. (2018). Nivolumab plus ipilimumab or nivolumab alone versus ipilimumab alone in advanced melanoma (CheckMate 067): 4-year outcomes of a multicentre, randomised, phase 3 trial. *Lancet Oncol* 19(11)**,** 1480-1492.

Hsu, C., Lee, S.-H., Ejadi, S., Even, C., Cohen, R.B., Le Tourneau, C., et al. (2017). Safety and antitumor activity of pembrolizumab in patients with programmed death-ligand 1–positive nasopharyngeal carcinoma: Results of the KEYNOTE-028 study. *J Clin Oncol* 35(36)**,** 4050-4056.

Huang, J., Mo, H., Zhang, W., Chen, X., Qu, D., Wang, X., et al. (2019). Promising efficacy of SHR‐1210, a novel anti–programmed cell death 1 antibody, in patients with advanced gastric and gastroesophageal junction cancer in China. *Cancer* 125(5)**,** 742-749.

Huang, J., Xu, B., Mo, H., Zhang, W., Chen, X., Wu, D., et al. (2018). Safety, activity, and biomarkers of SHR-1210, an anti-PD-1 antibody, for patients with advanced esophageal carcinoma. *Clin Cancer Res* 24(6)**,** 1296-1304.

Hui, R., Garon, E., Goldman, J., Leighl, N., Hellmann, M., Patnaik, A., et al. (2017). Pembrolizumab as first-line therapy for patients with PD-L1-positive advanced non-small cell lung cancer: a phase 1 trial. *Ann Oncol* 28(4)**,** 874-881.

Kang, Y.-K., Boku, N., Satoh, T., Ryu, M.-H., Chao, Y., Kato, K., et al. (2017). Nivolumab in patients with advanced gastric or gastro-oesophageal junction cancer refractory to, or intolerant of, at least two previous chemotherapy regimens (ONO-4538-12, ATTRACTION-2): a randomised, double-blind, placebo-controlled, phase 3 trial. *Lancet* 390(10111)**,** 2461-2471.

Kaufman, H.L., Russell, J., Hamid, O., Bhatia, S., Terheyden, P., D'Angelo, S.P., et al. (2016). Avelumab in patients with chemotherapy-refractory metastatic Merkel cell carcinoma: a multicentre, single-group, open-label, phase 2 trial. *Lancet Oncol* 17(10)**,** 1374-1385.

Keilholz, U., Mehnert, J.M., Bauer, S., Bourgeois, H.P., Patel, M.R., Gravenor, D., et al. (2018). Avelumab in patients with previously treated metastatic melanoma: Phase 1b results from the JAVELIN Solid Tumor trial. *J Immunother Cancer* 7(1)**,** 12.

Kudo, T., Hamamoto, Y., Kato, K., Ura, T., Kojima, T., Tsushima, T., et al. (2017). Nivolumab treatment for oesophageal squamous-cell carcinoma: an open-label, multicentre, phase 2 trial. *Lancet Oncol* 18(5)**,** 631-639.

Le Tourneau, C., Hoimes, C., Zarwan, C., Wong, D.J., Bauer, S., Claus, R., et al. (2018). Avelumab in patients with previously treated metastatic adrenocortical carcinoma: phase 1b results from the JAVELIN solid tumor trial. *J Immunother Cancer* 6(1)**,** 111.

Lisberg, A., Cummings, A., Goldman, J., Bornazyan, K., Reese, N., Wang, T., et al. (2018). A phase II study of pembrolizumab in EGFR-mutant, PD-L1+, tyrosine kinase inhibitor naïve patients with advanced NSCLC. *J Thorac Oncol* 13(8)**,** 1138-1145.

Lukas, R.V., Rodon, J., Becker, K., Wong, E.T., Shih, K., Touat, M., et al. (2018). Clinical activity and safety of atezolizumab in patients with recurrent glioblastoma. *J Neurooncol* 140(2)**,** 317-328.

Maruyama, D., Hatake, K., Kinoshita, T., Fukuhara, N., Choi, I., Taniwaki, M., et al. (2017). Multicenter phase II study of nivolumab in Japanese patients with relapsed or refractory classical Hodgkin lymphoma. *Cancer Sci* 108(5)**,** 1007-1012.

Mehnert, J.M., Varga, A., Brose, M.S., Aggarwal, R.R., Lin, C.-C., Prawira, A., et al. (2019). Safety and antitumor activity of the anti–PD-1 antibody pembrolizumab in patients with advanced, PD-L1–positive papillary or follicular thyroid cancer. *BMC Cancer* 19(1)**,** 196.

Mizugaki, H., Yamamoto, N., Murakami, H., Kenmotsu, H., Fujiwara, Y., Ishida, Y., et al. (2016). Phase I dose-finding study of monotherapy with atezolizumab, an engineered immunoglobulin monoclonal antibody targeting PD-L1, in Japanese patients with advanced solid tumors. *Invest New Drugs* 34(5)**,** 596-603.

Mo, H., Huang, J., Xu, J., Chen, X., Wu, D., Qu, D., et al. (2018). Safety, anti-tumour activity, and pharmacokinetics of fixed-dose SHR-1210, an anti-PD-1 antibody in advanced solid tumours: a dose-escalation, phase 1 study. *Br J Cancer* 119(5)**,** 538.

Morris, V.K., Salem, M.E., Nimeiri, H., Iqbal, S., Singh, P., Ciombor, K., et al. (2017). Nivolumab for previously treated unresectable metastatic anal cancer (NCI9673): a multicentre, single-arm, phase 2 study. *Lancet Oncol* 18(4)**,** 446-453.

Muro, K., Chung, H.C., Shankaran, V., Geva, R., Catenacci, D., Gupta, S., et al. (2016). Pembrolizumab for patients with PD-L1-positive advanced gastric cancer (KEYNOTE-012): a multicentre, open-label, phase 1b trial. *Lancet Oncol* 17(6)**,** 717-726.

Nanda, R., Chow, L., Dees, E.C., Berger, R., Gupta, S., Geva, R., et al. (2016). Pembrolizumab in patients with advanced triple-negative breast cancer: phase Ib KEYNOTE-012 study. *J Clin Oncol* 34(21)**,** 2460-2467.

Nishio, M., Hida, T., Atagi, S., Sakai, H., Nakagawa, K., Takahashi, T., et al. (2017). Multicentre phase II study of nivolumab in Japanese patients with advanced or recurrent non-squamous non-small cell lung cancer. *ESMO Open* 2(Suppl 1)**,** e000108.

Nishio, M., Takahashi, T., Yoshioka, H., Nakagawa, K., Fukuhara, T., Yamada, K., et al. (2019). KEYNOTE‐025: Phase 1b study of pembrolizumab in Japanese patients with previously treated programmed death ligand 1–positive advanced non–small‐cell lung cancer. *Cancer Sci* 110(3)**,** 1012.

O’Neil, B.H., Wallmark, J.M., Lorente, D., Elez, E., Raimbourg, J., Gomez-Roca, C., et al. (2017). Safety and antitumor activity of the anti–PD-1 antibody pembrolizumab in patients with advanced colorectal carcinoma. *PLoS One* 12(12)**,** e0189848.

Ott, P., Piha-Paul, S., Munster, P., Pishvaian, M., Van Brummelen, E., Cohen, R., et al. (2017). Safety and antitumor activity of the anti-PD-1 antibody pembrolizumab in patients with recurrent carcinoma of the anal canal. *Ann Oncol* 28(5)**,** 1036-1041.

Overman, M.J., McDermott, R., Leach, J.L., Lonardi, S., Lenz, H.-J., Morse, M.A., et al. (2017). Nivolumab in patients with metastatic DNA mismatch repair-deficient or microsatellite instability-high colorectal cancer (CheckMate 142): an open-label, multicentre, phase 2 study. *Lancet Oncol* 18(9)**,** 1182-1191.

Patel, M.R., Ellerton, J., Infante, J.R., Agrawal, M., Gordon, M., Aljumaily, R., et al. (2018). Avelumab in metastatic urothelial carcinoma after platinum failure (JAVELIN Solid Tumor): pooled results from two expansion cohorts of an open-label, phase 1 trial. *Lancet Oncol* 19(1)**,** 51-64.

Peters, S., Gettinger, S., Johnson, M.L., Jänne, P.A., Garassino, M.C., Christoph, D., et al. (2017). Phase II trial of atezolizumab as first-line or subsequent therapy for patients with programmed death-ligand 1–selected advanced non–small-cell lung cancer (BIRCH). *J Clin Oncol* 35(24)**,** 2781.

Plimack, E.R., Bellmunt, J., Gupta, S., Berger, R., Chow, L.Q., Juco, J., et al. (2017). Safety and activity of pembrolizumab in patients with locally advanced or metastatic urothelial cancer (KEYNOTE-012): a non-randomised, open-label, phase 1b study. *Lancet Oncol* 18(2)**,** 212-220.

Powles, T., Durán, I., Van Der Heijden, M.S., Loriot, Y., Vogelzang, N.J., De Giorgi, U., et al. (2018). Atezolizumab versus chemotherapy in patients with platinum-treated locally advanced or metastatic urothelial carcinoma (IMvigor211): a multicentre, open-label, phase 3 randomised controlled trial. *Lancet* 391(10122)**,** 748-757.

Powles, T., O'donnell, P.H., Massard, C., Arkenau, H.-T., Friedlander, T.W., Hoimes, C.J., et al. (2017). Efficacy and safety of durvalumab in locally advanced or metastatic urothelial carcinoma: updated results from a phase 1/2 open-label study. *JAMA Oncol* 3(9)**,** e172411-e172411.

Pujol, J.-L., Greillier, L., Audigier-Valette, C., Moro-Sibilot, D., Uwer, L., Hureaux, J., et al. (2019). A Randomized Non-Comparative Phase 2 Study of Anti-Programmed Cell Death-Ligand 1 Atezolizumab or Chemotherapy as Second-Line Therapy in Patients with Small Cell Lung Cancer: Results from the IFCT-1603 Trial. *J Thorac Oncol***,** doi: 10.1016/j.jtho.2019.1001.1008.

Quispel-Janssen, J., van der Noort, V., de Vries, J.F., Zimmerman, M., Lalezari, F., Thunnissen, E., et al. (2018). Programmed death 1 blockade with nivolumab in patients with recurrent malignant pleural mesothelioma. *J Thorac Oncol* 13(10)**,** 1569-1576.

Reck, M., Rodríguez-Abreu, D., Robinson, A.G., Hui, R., Csőszi, T., Fülöp, A., et al. (2016). Pembrolizumab versus chemotherapy for PD-L1–positive non–small-cell lung cancer. *N Engl J Med* 375(19)**,** 1823-1833.

Rittmeyer, A., Barlesi, F., Waterkamp, D., Park, K., Ciardiello, F., Von Pawel, J., et al. (2017). Atezolizumab versus docetaxel in patients with previously treated non-small-cell lung cancer (OAK): a phase 3, open-label, multicentre randomised controlled trial. *Lancet* 389(10066)**,** 255-265.

Rizvi, N.A., Mazières, J., Planchard, D., Stinchcombe, T.E., Dy, G.K., Antonia, S.J., et al. (2015). Activity and safety of nivolumab, an anti-PD-1 immune checkpoint inhibitor, for patients with advanced, refractory squamous non-small-cell lung cancer (CheckMate 063): a phase 2, single-arm trial. *Lancet Oncol* 16(3)**,** 257-265.

Robert, C., Ribas, A., Wolchok, J.D., Hodi, F.S., Hamid, O., Kefford, R., et al. (2014). Anti-programmed-death-receptor-1 treatment with pembrolizumab in ipilimumab-refractory advanced melanoma: a randomised dose-comparison cohort of a phase 1 trial. *Lancet* 384(9948)**,** 1109-1117.

Rosenberg, J.E., Hoffman-Censits, J., Powles, T., Van Der Heijden, M.S., Balar, A.V., Necchi, A., et al. (2016). Atezolizumab in patients with locally advanced and metastatic urothelial carcinoma who have progressed following treatment with platinum-based chemotherapy: a single-arm, multicentre, phase 2 trial. *Lancet* 387(10031)**,** 1909-1920.

Schachter, J., Ribas, A., Long, G.V., Arance, A., Grob, J.-J., Mortier, L., et al. (2017). Pembrolizumab versus ipilimumab for advanced melanoma: final overall survival results of a multicentre, randomised, open-label phase 3 study (KEYNOTE-006). *Lancet* 390(10105)**,** 1853-1862.

Scherpereel, A., Mazieres, J., Greillier, L., Lantuejoul, S., Dô, P., Bylicki, O., et al. (2019). Nivolumab or nivolumab plus ipilimumab in patients with relapsed malignant pleural mesothelioma (IFCT-1501 MAPS2): a multicentre, open-label, randomised, non-comparative, phase 2 trial. *Lancet Oncol* 20(2)**,** 239-253.

Shah, M.A., Kojima, T., Hochhauser, D., Enzinger, P., Raimbourg, J., Hollebecque, A., et al. (2018). Efficacy and safety of pembrolizumab for heavily pretreated patients with advanced, metastatic adenocarcinoma or squamous cell carcinoma of the esophagus: the phase 2 KEYNOTE-180 study. *JAMA Oncol***,** doi: 10.1001/jamaoncol.2018.5441.

Sharma, P., Callahan, M.K., Bono, P., Kim, J., Spiliopoulou, P., Calvo, E., et al. (2016). Nivolumab monotherapy in recurrent metastatic urothelial carcinoma (CheckMate 032): a multicentre, open-label, two-stage, multi-arm, phase 1/2 trial. *Lancet Oncol* 17(11)**,** 1590-1598.

Sharma, P., Retz, M., Siefker-Radtke, A., Baron, A., Necchi, A., Bedke, J., et al. (2017). Nivolumab in metastatic urothelial carcinoma after platinum therapy (CheckMate 275): a multicentre, single-arm, phase 2 trial. *Lancet Oncol* 18(3)**,** 312-322.

Shimizu, T., Seto, T., Hirai, F., Takenoyama, M., Nosaki, K., Tsurutani, J., et al. (2016). Phase 1 study of pembrolizumab (MK-3475; anti-PD-1 monoclonal antibody) in Japanese patients with advanced solid tumors. *Invest New Drugs* 34(3)**,** 347-354.

Shitara, K., Özgüroğlu, M., Bang, Y.-J., Di Bartolomeo, M., Mandalà, M., Ryu, M.-H., et al. (2018). Pembrolizumab versus paclitaxel for previously treated, advanced gastric or gastro-oesophageal junction cancer (KEYNOTE-061): a randomised, open-label, controlled, phase 3 trial. *Lancet* 392(10142)**,** 123-133.

Siu, L.L., Even, C., Mesía, R., Remenar, E., Daste, A., Delord, J.-P., et al. (2019). Safety and Efficacy of Durvalumab With or Without Tremelimumab in Patients With PD-L1–Low/Negative Recurrent or Metastatic HNSCC: The Phase 2 CONDOR Randomized Clinical Trial. *Lancet Oncol* 5(2)**,** 195-203.

Tahara, M., Muro, K., Hasegawa, Y., Chung, H.C., Lin, C.C., Keam, B., et al. (2018). Pembrolizumab in Asia‐Pacific patients with advanced head and neck squamous cell carcinoma: Analyses from KEYNOTE‐012. *Cancer Sci* 109(3)**,** 771-776.

Tawbi, H.A., Burgess, M., Bolejack, V., Van Tine, B.A., Schuetze, S.M., Hu, J., et al. (2017). Pembrolizumab in advanced soft-tissue sarcoma and bone sarcoma (SARC028): a multicentre, two-cohort, single-arm, open-label, phase 2 trial. *Lancet Oncol* 18(11)**,** 1493-1501.

Topalian, S.L., Sznol, M., McDermott, D.F., Kluger, H.M., Carvajal, R.D., Sharfman, W.H., et al. (2014). Survival, durable tumor remission, and long-term safety in patients with advanced melanoma receiving nivolumab. *J Clin Oncol* 32(10)**,** 1020.

Varga, A., Piha-Paul, S., Ott, P.A., Mehnert, J.M., Berton-Rigaud, D., Morosky, A., et al. (2019). Pembrolizumab in patients with programmed death ligand 1–positive advanced ovarian cancer: Analysis of KEYNOTE-028. *Gynecol Oncol* 152(2)**,** 243-250.

Weber, J.S., D'Angelo, S.P., Minor, D., Hodi, F.S., Gutzmer, R., Neyns, B., et al. (2015). Nivolumab versus chemotherapy in patients with advanced melanoma who progressed after anti-CTLA-4 treatment (CheckMate 037): a randomised, controlled, open-label, phase 3 trial. *Lancet Oncol* 16(4)**,** 375-384.

Yamazaki, N., Kiyohara, Y., Uhara, H., Uehara, J., Fujimoto, M., Takenouchi, T., et al. (2017). Efficacy and safety of nivolumab in Japanese patients with previously untreated advanced melanoma: a phase II study. *Cancer Sci* 108(6)**,** 1223-1230.

Younes, A., Santoro, A., Shipp, M., Zinzani, P.L., Timmerman, J.M., Ansell, S., et al. (2016). Nivolumab for classical Hodgkin's lymphoma after failure of both autologous stem-cell transplantation and brentuximab vedotin: a multicentre, multicohort, single-arm phase 2 trial. *Lancet Oncol* 17(9)**,** 1283-1294.

Zinzani, P.L., Ribrag, V., Moskowitz, C.H., Michot, J.-M., Kuruvilla, J., Balakumaran, A., et al. (2017). Safety and tolerability of pembrolizumab in patients with relapsed/refractory primary mediastinal large B-cell lymphoma. *Blood* 130(3)**,** 267-270.
